# Sophisticated Interfaces Between Biosensors and Organoids: Advancing Towards Intelligent Multimodal Monitoring Physiological Parameters

**DOI:** 10.3390/bios15090557

**Published:** 2025-08-22

**Authors:** Yuqi Chen, Shuge Liu, Yating Chen, Miaomiao Wang, Yage Liu, Zhan Qu, Liping Du, Chunsheng Wu

**Affiliations:** 1 Institute of Medical Engineering, Department of Biophysics, School of Basic Medical Sciences, Health Science Center, Xi’an Jiaotong University, Xi’an 710061, China; chen-yuqi@stu.xjtu.edu.cn (Y.C.); 4120115012@stu.xjtu.edu.cn (S.L.); ytc20201011@stu.xjtu.edu.cn (Y.C.); wmm15029418463@stu.xjtu.edu.cn (M.W.); liuyage@stu.xjtu.edu.cn (Y.L.); 2 Key Laboratory of Environment and Genes Related to Diseases (Xi’an Jiaotong University), Ministry of Education of China, Xi’an 710061, China

**Keywords:** biosensors, organoids, multimodal technology, microfluidics, electrochemical sensors, microelectrode arrays, nanomaterials

## Abstract

The integration of organoids with biosensors serves as a miniaturized model of human physiology and diseases, significantly transforming the research frameworks surrounding drug development, toxicity testing, and personalized medicine. This review aims to provide a comprehensive framework for researchers to identify suitable technical approaches and to promote the advancement of organoid sensing towards enhanced biomimicry and intelligence. To this end, several primary methods for technology integration are systematically outlined and compared, which include microfluidic integrated systems, microelectrode array (MEA)-based electrophysiological recording systems, optical sensing systems, mechanical force sensing technologies, field-effect transistor (FET)-based sensing techniques, biohybrid systems based on synthetic biology tools, and label-free technologies, including impedance, surface plasmon resonance (SPR), and mass spectrometry imaging. Through multimodal collaboration such as the combination of MEA for recording electrical signals from cardiac organoids with micropillar arrays for monitoring contractile force, these technologies can overcome the limitations inherent in singular sensing modalities and enable a comprehensive analysis of the dynamic responses of organoids. Furthermore, this review discusses strategies for integrating strategies of multimodal sensing approaches (e.g., the combination of microfluidics with MEA and optical methods) and highlights future challenges related to sensor implantation in vascularized organoids, signal stability during long-term culture, and the standardization of clinical translation.

## 1. Introduction

In vitro experiments and animal experiments serve as critical methodologies for disease modeling and drug screening [1]. Nonetheless, the existing in vitro and animal studies utilized in drug discovery often fail to replicate the intricate nature of human systems and organs, which can lead to inaccurate predication of drug toxicity and, consequently, a high rate of failure in clinical trials [2]. Organoids, which are three-dimensional cellular structures and contribute to the functionality of specific organs or tissues, are generated by inducing the differentiation of stem cells or organ progenitor cells through three-dimensional culture techniques in vitro. These organoids exhibit stable phenotypic and genetic traits, allowing for prolonged culture in vitro [3]. Due to their miniature three-dimensional cellular aggregates that retain essential structural and functional characteristics of actual organs, organoids are better equipped to represent the properties of human tissues [4]. They introduce a new research paradigm for clinical investigations and may serve as a valuable adjunct to traditional preclinical cell culture techniques and in vivo animal studies in the short term. In the long term, they have the potential to serve as viable alternatives [5].

**Figure 1:**
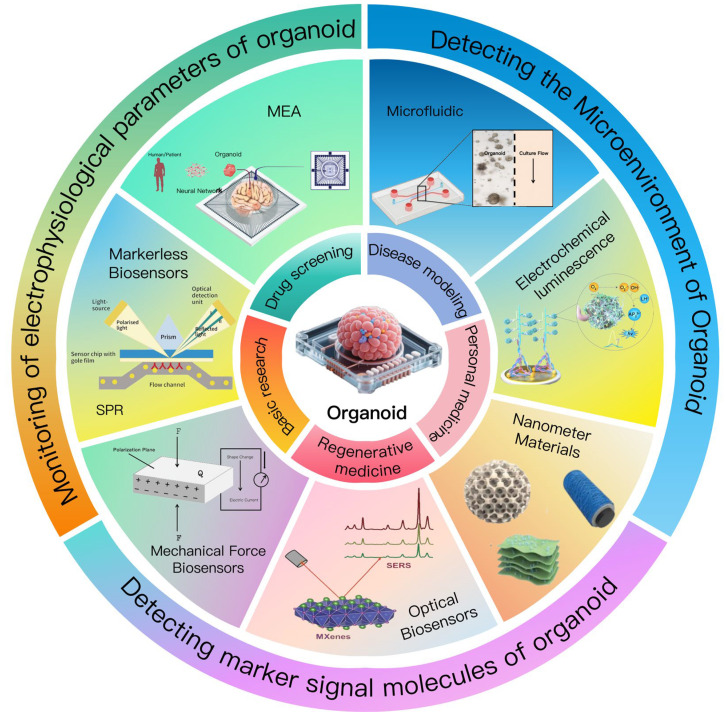
Overview of organoid research and application fields: technology and application map from basic research to translational medicine.

The rapid advancement of biosensor technology has facilitated the integration of organoids and biosensors, presenting a new approach for the real-time monitoring of physiological parameters (Figure 1). The applications of biosensors have the potential to enhance the early diagnosis and treatment of diseases by monitoring changes in biomarkers in vivo [6,7]. This convergence has led to the emergence of the innovative research field known as “organoids-biosensors”. Electrochemical sensors provide real-time insights into the functional status of organoids by detecting variations in the electrical signals associated with metabolic molecules, such as glucose and lactate [8]. Additionally, microelectrode arrays enable the recording of electrophysiological activities in neural and myocardial organoids with high spatiotemporal resolution [9,10], thereby elucidating the mechanisms underlying cellular network interactions. Furthermore, optical sensors employing fluorescence imaging and Raman spectroscopy [11] facilitate the analysis of metabolic pathways and structural changes in organoids at the molecular level. The incorporation of these technologies has transitioned organoid research from static observation to dynamic functional analysis [12], thereby offering quantitative data that supports a deeper understanding of organ development and the progression of diseases.

In this context, the advent of multimodal technology has improved the comprehensiveness and accuracy of monitoring processes [13]. The convergence of multimodal technologies has further expanded the dimension of organoid monitoring [14]. By integrating microfluidic perfusion systems, mechanical sensing, and advanced intelligent algorithms [15,16], researchers were able to simultaneously obtain the hydrodynamic parameters, mechanical stress responses, and electrophysiological signals from organoids, thereby enabling the construction of multidimensional data models. This cross-scale monitoring system not only improves the accuracy of drug screening, but also establish a technical foundation for the clinical application of “organoid-on-a-chip” technologies in personalized medicine [17]. The transition from single-parameter detection to multi-dimensional collaborative analysis signifies that the intersection of organoids and biosensors is accelerating its evolution in the direction of greater integration and intelligence [18,19].

## 2. Organoids and Biosensors

The integration of organoids with biosensors has emerged as a transformative approach to decode the complex physiological and functional dynamics of these 3D cellular constructs, bridging the gap between static observation and real-time, quantitative analysis. Section 2 focuses on the diverse array of biosensing technologies tailored to monitor organoids, encompassing three core dimensions: the microenvironmental cues (e.g., metabolites, ions, and mechanical forces) that regulate organoid development, the electrophysiological activities that reflect functional maturity (particularly in neural and cardiac organoids), and the signaling molecules (e.g., biomarkers, metabolites) that indicate cellular states and responses. By exploring technologies such as microfluidic systems, electrochemical sensors, microelectrode arrays (MEAs), optical sensors, and label-free techniques, this section elucidates how biosensors enable precise, dynamic, and multi-parametric monitoring of organoids, thereby advancing our understanding of their biology and accelerating their applications in drug screening, disease modeling, and personalized medicine.

### 2.1. Organoid Microenvironment Monitoring Sensors

#### 2.1.1. Microfluidic System

**Figure 2:**
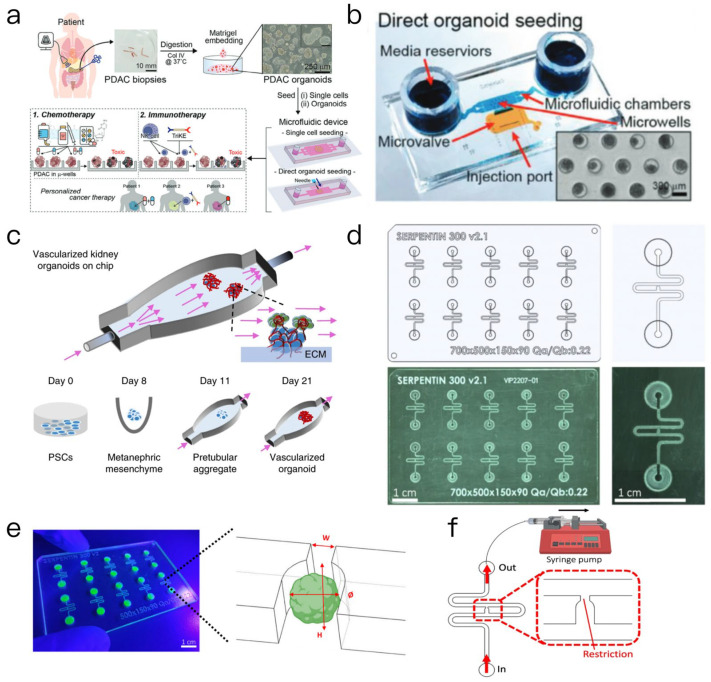
(**a**) A single-cell seeding microfluidic device with 5 independent chambers featuring a microwell array [25]. (**b**) Organoids are inoculated through lateral injection ports by applying negative pressure [25]. (**c**) Renal organoids are cultured on an engineered ECM with controlled fluid shear stress [27]. (**d**) A computer-aided designed microfluidic chip [28]. (**e**) U-shaped trapping regions for capturing organoids [28]. (**f**) Top view of the microfluidic device. A syringe pump was connected to the outlet of the channel to introduce fluid perfusion [28].

Organoid-on-a-chip technology represents an advancement in the field of biotechnology, specifically in the simulation of human organ functions through the three-dimensional culture of cells utilizing in vitro microarray technology and microfluidic chip technologies [20]. This innovative approach operates at the microenvironmental level, allowing for the regulation of fluid perfusion, mechanical tension, and chemical gradient, thereby enhancing the biomimetic characteristics and functional maturity of organ structures. Compared to traditional organoids, microarray technology offers a more precise simulation of physiological organ activities, such as blood flow and respiration, while also integrating sensors for real-time monitoring of cellular responses [21]. In oncological research, this technology effectively replicates the tumor microenvironment and metastatic processes, providing a more physiologically relevant alternative for drug testing and mechanistic research, thus surpassing traditional cancer models [22]. The microfluidic platform facilitates the efficient screening of drug compounds [23], especially in the application of anti-cancer drugs, thereby advancing the discovery of novel pharmacological agents. For example, microfluidic chip-based drug screening can emulate drug metabolism processes within the human body, allowing for more accurate predictions regarding the biological effects and toxicity of various drugs [24]. A specific application of this technology involved the development of a microfluidic system designed for personalized chemotherapy and immunotherapy testing using pancreatic cancer biopsy tissues [25]. In a previous reported study, researchers obtained needle biopsy samples from patients, which were subsequently digested and cultured into organoids. These organoids were then placed into two distinct microfluidic devices: Type 1 for single-cell seeding and Type 2 for the direct implantation of intact organoids or fragments through a side injection port (Figure 2a,b). This approach addresses the problem of insufficient cell numbers typically encountered in traditional culture methods. To evaluate the efficacy of drug screening within this microfluidic device, various agents, including encorafenib (a BRAF inhibitor), binimetinib (a MEK inhibitor), their combinations, and the first-line chemotherapy drug Gemcitabine, were administered. The findings indicated that the microfluidic organoids accurately reflected drug responses, exemplified by the significant cytotoxic effect of binimetinib monotherapy on BRAF mutant organoids. The study confirmed that the microfluidic organoids exhibited phenotypic and genotypic similarities to the gold standard Matrigel organoids, while also demonstrating advantages such as spheroid uniformity, reduced dependence on cell numbers, and the elimination of Matrigel requirements. The integration of chemotherapy drug testing with natural killer (NK) cells and new biologics for immunotherapy evaluation provides a promising platform for personalized cancer treatment based on cancer biopsy analysis, with the potential to evolve into a companion diagnostic tool for chemotherapy or immunotherapy.

The emergency of microfluidic technology provides new opportunities for the culture and research of organoids. The design and optimization of microfluidic chips is the key to achieving effective organoid development. First, fluid dynamics simulations serve as a valuable tool for researchers to comprehend fluid behavior of fluids within microfluidic systems, which is vital for optimizing the distribution of nutrients and growth factors in organoid cultures [26]. The establishment of vascularized organ-on-a-chip systems in tissue engineering has historically posed significant challenges. Conventional microfluidic chips often fail to accurately replicate in vivo flow conditions, and achieving functional vascularization of induced pluripotent stem cell (iPSC)-derived organoids cultured in vitro remains a challenge. This article outlines a methodology for culturing kidney organoids on an in vitro microfluidic chip. In a three-dimensional (3D)-printed microfluidic chip, organoids are perfused with controlled wall shear stresses (i.e., fluid shear stresses), facilitating their attachment to a gel–brain extracellular matrix (Figure 2c). This method expands the pool of endogenous endothelial progenitor cells, generates a perfusable luminal vascular network encircled by parietal cells, and enable vascularized renal organoids to develop more mature podocytes and tubular compartments. This maturation process improves cell polarity and adult gene expression, with glomerular vascular development resembling that of embryonic mammalian kidneys. Furthermore, endogenous vascular endothelial growth factor (VEGF) gradients are critical for the integration of blood vessels with these compartments. This innovative approach paves the way for advanced studies on kidney development, disease, and regeneration [27]. Similarly, researchers have developed a new microfluidic platform for the establishment and monitoring endothelial network formation on a chip, as well as for the functional connectivity of blood organoid chips, thereby promoting the growth, maturation, and functionality of organoid systems. This article also describes a microfluidic platform designed for the construction of functional vascularized organoids [28]. The microfluidic chip is composed of cyclic olefin copolymers (COCs), which offer long-term stability and favorable optical properties, and features ten independent microchannels, each equipped with a trap site (Figure 2d) that can be adjusted according to the size of the organoids. For instance, vascular organoids with a diameter of approximately 600 μm correspond to traps that are 300 μm wide and 800 μm high, while mesenchymal and pancreatic islet spheres with a diameter of around 300 μm correspond to traps that are 200 μm wide and 400 μm high (Figure 2e). The study demonstrates that the chip operates in parallel with a ten-channel syringe pump, allowing for precise control of fluid priming. Based on the Landau-Levich-Bretherton effect, a thin hydrogel layer remains on the microchannel wall, which promotes the self-organization of endothelial cells into a vascular network that traverses the trap site, thereby providing nutrient perfusion for the organoids (Figure 2f). The platform successfully realized the vascularization of mesenchymal spheroids, vascular organoids and pancreatic islet spheroids on the chip for duration of up to 30 days, significantly enhancing the growth, maturation and functionality of the organoids, and providing an effective model for the construction of functional vascularized tissues in vitro.

Microfluidics has emerged as a significant tool for the real-time monitoring of the organoid culture environments [29]. By integrating sensors, microfluidic chips are capable of continuously tracking essential parameters such as pH, partial pressure of oxygen, and nutrient concentrations within the culture medium. For example, gas diffusion techniques can be used to establish stable oxygen gradients within microfluidic systems [30], thereby simulating varying oxygen levels characteristic of the tumor microenvironment. This approach facilitates the investigation of cellular proliferation and migration behaviors, demonstrating considerable promise in the fields of tumor biology and pharmacological development. The extracellular matrix (ECM) is vital for preserving tissue architecture and functionality [31], with the orientation and arrangement of collagen fibers influencing the directionality and velocity of cellular movement. Microfluidic devices enable the precise layered deposition of diverse components within a controlled microenvironment, thereby allowing for the reconstruction of the original structural and functional attributes of tissues [32]. In recent years, the application of microfluidic technology in drug metabolism research has advanced rapidly, especially in the simulation of liver and kidney functions [33,34]. Microfluidic technology provides a more reliable model for assessing drug toxicity through the development of body-on-a-chip or organ-on-a-chip systems, which enables the simulation of the physiological environment of human organs in vitro [35].

#### 2.1.2. Electrochemical Biosensors

Electrochemical sensors detect and quantify biomarkers based on the principles of electrochemical reactions [36], such as glucose and lactic acid. These sensors typically indicate the concentration of target molecules through changes in the current or voltage of the electrode. For example, glucose sensors usually utilize enzymes (e.g., glucose oxidase) to catalyze reactions, generating current changes that are proportional to the glucose concentration. Similarly, lactic acid sensors may monitor changes in lactic acid through electrochemical reactions, which is particularly important in metabolic research and exercise physiology [37].

In addition, in recent years, the design of electrochemical sensors has gradually advanced towards new materials and novel sensor platforms. For instance, electrochemical sensors using new two-dimensional materials like MXene have demonstrated excellent electrochemical performance and sensitivity, enabling effective detection of specific biomarkers in complex biological environments [38]. These advancements have expanded the application potential of electrochemical sensors in organoid monitoring, enhancing the capability for real-time monitoring of physiological states. Electrochemical sensors face challenges related to sensitivity and selectivity in complex biological environments, such as organoid culture media [3]. Strategies to improve sensitivity include the use of nanomaterials with high surface areas, such as carbon nanotubes and graphene, which can significantly enhance the electrochemical reaction rate of the electrode [39], thereby improving the response capability of the sensor. Furthermore, optimizing the surface chemical properties of the electrode, such as through functionalization or modification of the electrode surface, can enhance the specificity of the sensor for target molecules [40] and reduce the impact of interfering substances.

Molecularly imprinted polymer (MIP) technology is often used for the development of electrochemical sensors due to its selectivity advantages [41]. This technology enables the construction of specific recognition sites on the electrode surface, thereby enhancing the sensor’s selectivity for the target analyte. For example, in the case of glucose sensors, MIP ensures that the sensor is responsive exclusively to glucose molecules, while significantly minimizing interference from structurally similar compounds, such as other sugars [42]. In addition, the incorporation of signal amplification techniques, such as the utilization of organic electrochemical transistors (OECTs) as amplifiers, can further improve the sensitivity of these sensors, enabling the detection of extremely low concentrations of biomarkers in practical applications [43].

The ongoing advancements in biomedical research have led to a significant trend towards the miniaturization and integration of electrochemical sensors [44]. This trend is anticipated to not only decrease costs, but also improve the portability and real-time monitoring capabilities of these sensors. The development of miniaturized sensors often relies on sophisticated manufacturing techniques, such as 3D printing and microfabrication, which enable sensors to work efficiently within confined spaces [45]. Within organoid culture systems, the integrated design of the electrochemical sensors enables the real-time monitoring of physiological parameters in the culture medium. For example, integrated sensors can be directly embedded into the culture medium, allowing for the continuous assessment of the metabolic status of organoids by monitoring changes in glucose and lactate levels. This integrated methodology not only improves the accuracy of monitoring, but also streamlines experimental procedure and minimizes the necessity for sample handling, thereby mitigating potential interferences. For example, the research group of Professor Y. Shrike Zhang from Harvard Medical School designed a microfluidic system integrated with a renewable electrochemical affinity biosensor [46]. Its core goal is to achieve continuous, in situ, and non-invasive monitoring of soluble biomarkers in organ-on-a-chip. The device is mainly composed of an electrochemical microelectrode, a microfluidic chip, and a system integration module. The electrochemical microelectrode uses electrochemical active materials such as gold, and its surface can be automatically functionalized through the microfluidic system to immobilize biorecognition elements such as aptamers or antibodies, generating an electrical signal through specific binding to the target biomarker. The microfluidic chip contains microchannels, valves, and reservoirs, which can precisely control fluid delivery. The “functionalization–detection–regeneration” process is automatically completed through valve switching. In the functionalization stage, a solution of recognition elements is delivered to immobilize biorecognition molecules. In the detection stage, the culture medium is guided to flow through the electrode to achieve biomarker detection. In the regeneration stage, a desorbent is delivered to restore the detection ability of the electrode. The system integration module connects the microfluidic chip with the electrochemical detection module and the fluid driving device through a breadboard, and realizes fully automated control of the whole process by combining the detection circuit and data analysis code. It is applicable to scenarios such as drug screening, disease modeling, and personalized medicine research, providing a powerful tool for dynamically analyzing the functions of microtissues. In this review, we compare the advantages and disadvantages of Electrochemical sensors of key technical (Table 1).

In the forthcoming years, electrochemical sensors are anticipated to undergo further miniaturization and integration, amalgamating various sensing technologies and analytical capabilities. This advancement will facilitate the simultaneous monitoring of multiple physiological parameters, thereby providing more powerful tools for personalized medicine and precision treatment. Such developments are expected to foster the extensive application of biosensors in clinical settings, particularly in the realms of real-time monitoring and early diagnosis of diseases, which holds important application prospects.

#### 2.1.3. Field-Effect Transistors (FETs)

FETs have become ideal tools for monitoring the microenvironment of organoids due to their high sensitivity, rapid response, and miniaturization characteristics. The application of FET technology enables researchers to monitor changes in the microenvironment of organoids in real-time without interfering with their growth [47]. For example, FET sensors can be used to detect changes in the concentration of metabolites in organoid culture media, which is crucial for understanding cell-to-cell interactions and the mechanisms of disease occurrence [48]. In addition, the high-throughput detection capability of FETs gives them unique advantages in drug screening and personalized therapy. They can quickly evaluate the effects of different drugs on organoids, thereby providing a more accurate basis for the formulation of clinical treatment plans [49,50]. FET sensors can not only effectively monitor the biochemical signals of organoids but also be combined with microfluidic technology to achieve more complex microenvironment simulation and real-time monitoring [51,52]. This combination not only improves the timeliness and accuracy of monitoring but also provides a new perspective for studying the physiological and pathological characteristics of organoids. A field-effect transistor (FET) is a semiconductor device that regulates current through an electric field, mainly consisting of a source, a drain, and a gate. Its working mechanism is based on adjusting the gate voltage to control the concentration of carriers in the channel, thereby affecting the magnitude of the channel current. In biosensing applications, the gate surface of FETs is usually modified with biomolecules. For example, biomolecules such as antibodies, enzymes, or DNA are immobilized to enable them to bind to target molecules. When the target molecule binds to the receptor, it changes the charge distribution on the gate surface, which in turn affects the conductivity of the channel. This mechanism allows FETs to achieve high sensitivity and rapid detection, and they are widely used in biosensors [53].

FETs are mainly classified into metal–oxide–semiconductor FETs (MOSFETs), organic FETs (OFETs), and nanowire FETs (NWFETs). Among them, NWFETs perform particularly well in the field of biosensing due to their high surface-area-to-volume ratio and low detection limit. NWFETs can provide higher sensitivity and faster response speed in sensor applications, especially suitable for the detection of biomolecules. This is because the small structure of nanowires can increase the contact area with target molecules, thereby improving the sensitivity and accuracy of detection. In addition, NWFETs also have the advantages of adjustability and easy combination with other nanomaterials, making them highly flexible in biosensor design [54,55].

To improve the selectivity of FETs in biosensors, various biofunctionalization strategies are usually adopted. These strategies include chemical modification and biomolecule immobilization. Among them, chemical modification methods such as silanization and gold nanoparticle modification can enhance the surface properties of FETs and their affinity for target molecules. In addition, by immobilizing biomolecules such as antibodies or aptamers, highly selective detection of specific biomarkers can be achieved. These functionalization strategies not only improve the sensitivity of the sensor but also enable efficient detection of target molecules in complex biological environments [56,57]. Moreover, studies on the combination of heterostructures of FETs and 2D materials also provide new development directions for their application in the field of biosensors and enhance their application potential [58,59]. The pH value of the organoid culture environment is an important factor affecting cell metabolism and proliferation. Studies have shown that changes in pH value can significantly affect the growth state and function of cells. For example, the suitable pH range is usually between 7.2 and 7.4, and the pH value within this range can promote cell proliferation and metabolic activity. If the pH value is too low or too high, it may inhibit cell growth and even cause cell apoptosis. The application of field-effect transistor (FET) technology, especially the use of H^+^-sensitive membranes (such as Ta_2_O_5_), can realize real-time monitoring of the pH value in the organoid culture environment. This monitoring method can not only timely feedback changes in the culture environment but also help researchers adjust the culture conditions to optimize the growth and function of organoids. By real-time monitoring of the pH value, researchers can obtain more accurate data, so as to make dynamic adjustments during the experiment to ensure that cells grow under optimal physiological conditions, thereby improving the success rate and repeatability of the experiment [60].

The monitoring of metabolites is one of the important means to evaluate the physiological state of organoids. During the culture of organoids, the dynamic changes of metabolites such as glucose and lactic acid can reflect the metabolic activity and growth state of cells. FETs modified with glucose oxidase can be used to realize real-time detection of glucose consumption in organoid metabolic activities. This method has the advantages of high sensitivity and fast reaction speed, which can timely reflect the utilization of glucose by cells. When cell activity increases, the consumption of glucose will increase significantly, which in turn leads to an increase in lactic acid production, and this process can be monitored in real-time by FETs. Studies have shown that changes in glucose metabolism are not only related to cell proliferation and apoptosis but may also affect the differentiation state of cells. Therefore, real-time monitoring of changes in the concentration of glucose and lactic acid provides an important basis for studying the physiological characteristics and pathological states of organoids, and helps to further understand the regulatory mechanism of cell metabolism [61,62].

The monitoring of ion concentrations, especially potassium ions (K^+^) and calcium ions (Ca^2+^), plays an important role in studying the electrophysiological activities of organoids. FETs prepared with ion–selective membranes (such as valinomycin modification) can be used for the detection of K^+^ concentration. K^+^ plays a key role in maintaining the membrane potential and the balance of the intracellular and extracellular environment, and changes in its concentration can directly affect the physiological activities of cells, including cell excitability and signal transduction. Therefore, real-time monitoring of changes in K^+^ concentration in organoids can not only reflect the electrophysiological state of cells but also provide important information for studying the mechanism of cell signal transduction. In addition, Ca^2+^, as an important medium for intracellular signal transduction, its concentration changes can also affect cell proliferation and differentiation. By monitoring the concentrations of K^+^ and Ca^2+^, we can more comprehensively understand the physiological state of organoids, thereby providing data support for the establishment of disease models and drug testing [63,64].

FET technology shows significant technical advantages in the field of biosensors, especially when applied in the monitoring of the organoid microenvironment. These advantages are mainly reflected in several aspects such as high sensitivity, rapid response, and miniaturization. Firstly, due to their high sensitivity, FET sensors can achieve a detection limit at the picomolar (pM) level, which makes them have great potential in biomolecule detection. For example, sensors using FET technology can achieve rapid and sensitive signal detection in complex biological environments, thereby effectively monitoring biomarkers in organoids [65]. Secondly, the rapid response characteristic of FET sensors allows them to complete data collection and processing within seconds, which is particularly important for real-time monitoring and dynamic analysis. This characteristic enables FET sensors to provide timely feedback in a rapidly changing biological environment, supporting real-time decision-making [66]. Finally, the miniaturized design of FETs makes them very suitable for high-throughput detection. This miniaturization not only improves the integration of the sensor but also reduces the production cost, which is conducive to large-scale application and promotion. In addition, the integrability of FETs and their compatibility with other microelectronic devices make them have broad application prospects in future intelligent medical devices [67].

### 2.2. Monitoring of Organoid Electrophysiological Parameters

#### 2.2.1. Microelectrode Array (MEA) Technology

MEA consists of numerous miniature electrodes capable of detecting electrical signals, such as action potential and field potentials, from neurons or cardiomyocytes with high throughput. These arrays facilitate the recording and stimulation of neural electrical activity, and are extensively utilized in brain–computer interface (BCI) applications, neuroscience research [68], and drug screening, among other areas. For example, MEAs are employed in studies related to epilepsy [69], the firing patterns of neurons in Parkinson’s disease, and the assessment of cardiomyocyte drug toxicity including evaluations of QT interval prolongation [70].

Brain organoids demonstrate electrical characteristics akin to those of the brain, exhibiting increased firing frequency and synchronization during development, which parallels the evolution of neural networks in vivo [71]. When neural organoids are cultured on MEAs, the electrodes can record their spontaneous electrical activity, thereby effectively reflecting the interconnections and network dynamics among neurons [9]. In contrast, cardiomyocytes derived from human heart organoids exhibit rhythmic contractions comparable to those of early human embryos, alongside action potentials characteristic of specific cardiac cell types, such as those found in the ventricles and atria, as well as nodular-like waveforms. Cardiac organoids successfully replicate the electrophysiological properties of cardiac tissue, thereby serving a crucial role in drug screening and regenerative medicine research [72].

MEA can be used to assess the electrophysiological properties and function of cardiomyocytes, contributing to the understanding of heart disease mechanisms and potential treatments. By recording the electrical activity of cardiomyocytes, researchers can analyze the heart’s electrical conduction system and its pharmacological responses, thereby providing new insights into the treatment of heart diseases [73]. In conclusion, the application of MEA technology in myocardial and neural organoids provides an important tool and platform for basic scientific research and clinical translation.

Conventional planar electrodes often encounter spatial constraints when interfacing with three-dimensional tissues, which can lead to suboptimal signal acquisition. Current electrophysiological recording techniques, such as patch-clamp, puncture microelectrodes, planar electrode arrays, and planar flexible electrodes, are inadequate for long-term suspension recording while preserving the morphology of organoids [74]. The implementation of a three-dimensional electrode structure can increase the contact area between the electrode and the organoids, thereby improving the quality and stability of the recorded signals.

**Figure 3:**
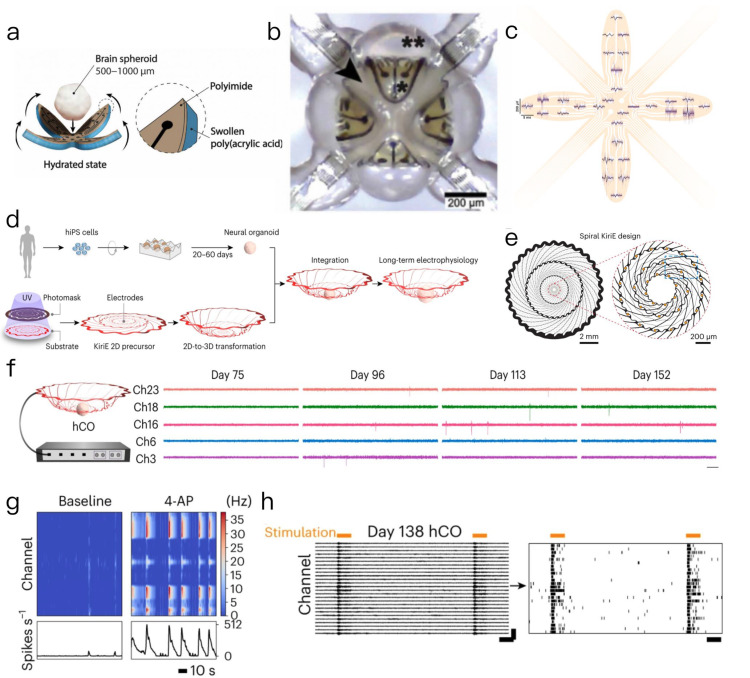
(**a**) Schematic: e-Flower reconfigures via PAA/Pi layer swelling difference; petals close to make electrodes attach to brain spheroids [75]. (**b**) Bright-field image of e-Flower wrapping brain spheroid, showing spheroid, petals, etc. Scale bar: 200 μm [75]. (**c**) 32-electrode 5 min recording of e-Flower; 2 channels silent, possibly far from active nerves [75]. (**d**) Schematic: Neural organoids from hiPS cells and integration with KiriE [76]. (**e**) Spiral KiriE connected by concentric rings, 32 electrodes in central area [76]. (**f**) 5-channel spontaneous activity maps of hCO at multiple differentiation days, scale bars 100 ms, 200 μV [76]. (**g**) Day 138 hCO: single-unit activity heatmaps before/after 4-AP [76]. (**h**) Day 138 hCO: light stimulation records and raster plots, showing stimulation duration [76].

In this context, we present a hydrogel-driven three-dimensional microelectrode array (3D MEA) referred to as the e-Flower. Its core design features a flower-shaped bilayer structure composed of a polyimide (PI) film and a polyacrylic acid (PAA) hydrogel. Specifically, the PI layer serves as the functional substrate, hosting 32 platinum microelectrodes (8 per petal) and electrical interconnects, while the PAA hydrogel is covalently grafted onto the backside of the PI petals (Figure 3a). This bilayer configuration is the key to its 2D-to-3D transformation. The actuation mechanism relies on the differential swelling properties of the two materials: when immersed in cell culture medium (CCM), the PAA hydrogel undergoes significant anisotropic swelling (expanding more in area and thickness) due to its hydrophilic network, whereas the PI layer remains dimensionally stable owing to its rigid polymer structure. This mismatch in swelling generates a bending torque, driving the initially flat PI petals to curl inward. The curvature of the petals is precisely tunable: by optimizing the PAA cross-linker concentration (1×, 0.05 wt% MBAA) and reswelling conditions (37 °C CCM), the e-Flower achieves a minimum bending radius of 300 μm—ideally matching the 500–1000 μm diameter range of cerebral spheroids. The encapsulation process unfolds in a time-dependent manner: when a preformed cerebral spheroid is placed at the center of the dry, flat e-Flower and CCM is introduced, the PAA hydrogel begins to swell within minutes. This triggers the petals to gradually close around the spheroid, with full 3D enclosure achieved in ~ 109 s (Figure 3b). The conformable contact between the electrodes and the spheroid surface is ensured by the hydrogel’s soft mechanics, avoiding mechanical damage to the fragile tissue. Electrophysiological recordings using a commercial MEA-2100 system demonstrated that the e-Flower can detect spontaneous neural activity across the spheroid surface. The 32 electrodes captured field potential waveforms with an average duration of ~2 ms (Figure 3c).

Notably, this process requires no external actuators or hazardous solvents, and the e-Flower seamlessly interfaces with commercial recording systems, offering a user-friendly “plug-and-play” solution for 3D neural tissue electrophysiology [75].

Similarly, Cui’s research team developed a geometrically cut ultra-thin electronic device named KiriE, which can self-folded from a two-dimensional pattern into a three-dimensional basket-like configuration (Figure 3d). This device incorporates 32 microelectrodes with a diameter of 1 cm and a total thickness of 0.9 μm at the center of 1 mm (Figure 3e). The platform demonstrated the capability to integrate with human cortical organoids (hCOs) over an extended period. Notably, no significant electrical activity was observed on day 75 of hCOs differentiation; however, a regular spike was recorded across multiple channels after day 96, with discharge frequency continuing to rise by day 152 (Figure 3f). When combined with optogenetic technology, the discharge frequency of hCOs increased from 0.24 ± 0.03 Hz to 5.44 ± 0.57 Hz through 590 nm photostimulation (Figure 3g). Additionally, the applications of the 4-aminopyridine (4AP) drug via a perfusion system resulted in an increase in discharge frequency from 0.49 ± 0.08 Hz to 1.60 ± 0.25 Hz (Figure 3h), while tetrodotoxin (TTX) almost completely inhibited discharge, thereby validating the platform’s regulatory response to neural activity [76].

Through the real-time monitoring of these electrical signals, researchers can gain valuable insights into the electrophysiological properties of cells within organoids and their mechanisms in response to external stimuli. These innovative 3D electrode technologies break through the spatial limitations imposed by traditional planar electrodes in the monitoring of 3D tissues, facilitating not only the recording of electrophysiological activities of organoids, but also enabling the long-term stable recording and precise regulation. This advancement provides unprecedented technical support for elucidating the dynamic functions of complex biological systems and their associated disease mechanisms, thereby promote further development of high-resolution and long-term monitoring in the field of “organoid-biosensors”.

Multimodal data fusion strategies are becoming increasingly important in the field of biomedical research. The advent of organ-on-a-chip technology has enabled researchers to effectively integrate mechanical stress loading with electrophysiological monitoring, thereby facilitating the quantification of alternations in the electrical activity of myocardial organoids and neural organoids. This approach aids in elucidating the influence of the mechanical environment on the function of neural networks [5,8]. A recent study using flexible MEAs to monitor the electrophysiological activity of myocardial organoids under dynamic loading conditions revealed that mechanical stress significantly enhanced the electrical activity of the cells. This observation suggests that mechanical stimulation may enhance the electrophysiological function of myocardial cells, which holds significant implications for understanding the pathological mechanisms underlying heart disease and its therapeutic interventions [77].

In recent years, graphene has emerged as an excellent nanomaterial capable of substantially reducing the interfacial impedance of electrodes by modification, thereby improving the electrochemical performance. One study demonstrated that graphene-based electrodes achieved a reduction in interfacial impedance of approximately 40% by increasing the efficiency of charge transport [78]. Nanoporous gold electrodes can provide a larger reaction interface, which makes the charge transfer process more efficient, thereby enhancing the response of the current signal [79]. In summary, MEA technology has shown great potential in the electrophysiological investigation of neural and myocardial organoids. Future development in this field will rely on interdisciplinary collaboration and continuous innovation in technology.

#### 2.2.2. Mechanical Force Transducers

Mechanical force sensors play a key role in biosensor and organoid research, particularly in monitoring the responses of biological tissues and organs to external mechanical stimuli [80]. Recent advancements in bioengineering technology have broadened the application of these sensors across various fields, including tumor biology and cardiovascular disease research [81], as well as the modeling of neurodegenerative diseases. In these contexts, mechanical force sensors facilitate the acquisition of real-time, continuous biophysical data, thereby elucidating the mechanical properties of cells and tissues under diverse physiological and pathological states.

Traditional biomechanical assessment methods often fail to accurately reflect the mechanical behavior of organoids in physiological states. In contrast, innovative technologies such as force sensors and micropillar arrays enable the real-time monitoring of organoid contraction and strain, yielding critical data for understanding cellular interactions with their microenvironment [82,83]. The development and function of organoids are not only affected by genetic factors, but also significantly regulated by the mechanical interactions within their microenvironment. Studies have shown that the growth of cells in a three-dimensional microenvironment is contingent upon factors such as matrix stiffness and fluid dynamics.

**Figure 4:**
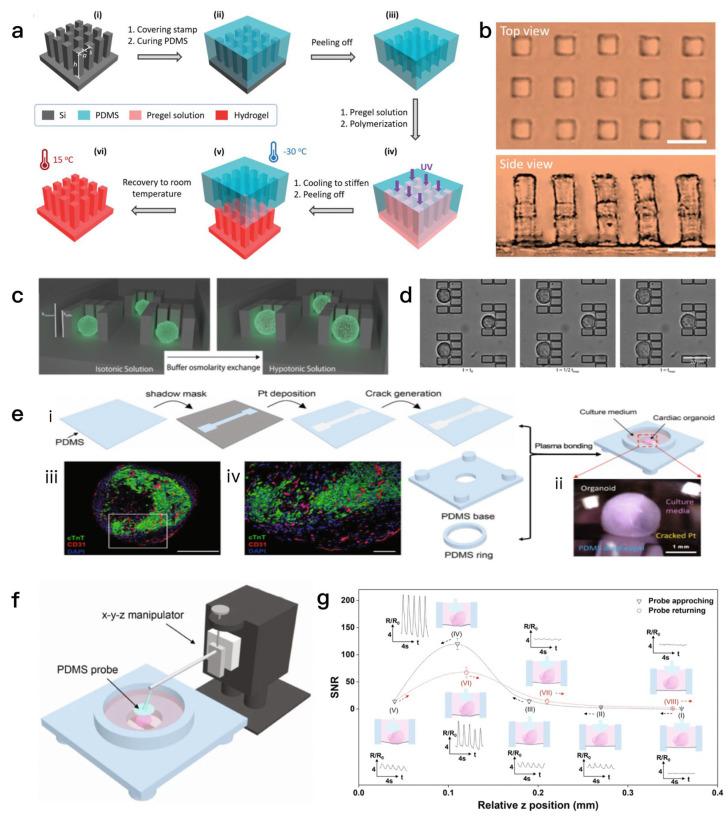
(**a**) Soft lithography process for hydrogel films with micropillars: PDMS molding, pregel UV polymerization, etc. [86]. (**b**) Micrograph of hydrogel film with cubic-arranged micropillars. Scale bar: 20 μm [86]. (**c**) Microfluidic CellHangars trap single cells to observe hypotonic swelling dynamics [88]. (**d**) Dynamic size changes of cells at different times after hypotonic buffer exchange [88]. (**e**) Fabrication of Pt-based nanocracked force-sensing diaphragm and its interface with organoids [90]. (**f**) Pt-based force-sensing diaphragm with probes monitors instantaneous organoid beating [90]. (**g**) Resistance changes of force-sensing diaphragm during “AFM-like” probe approaching/departing organoids [90].

These mechanical signals regulate cell behavior through mechanical transduction pathways, especially the YAP/TAZ signaling pathway, which is pivotal for promoting organoid formation and maturation [84]. As an emerging mechanical characterization tool, micropillar array technology offers high spatial resolution and accurate local force measurement capabilities. By quantifying the local forces exerted on the pillars through deflection monitoring, micropillar arrays provide valuable insights into the mechanical properties of cells and materials. For example, micropillar arrays have been shown to be useful for assessing the mechanical properties of cells [85] such as cell contractility and adhesion, which are essential for the cellular biological behavior and are subject to alterations in responses to changes in the microenvironment [86]. The study constructed a microcolumn array with a high aspect ratio on the surface of supramolecular hydrogels via soft lithography, solving the demolding problem caused by the weak mechanical properties of hydrogels. This hydrogel (poly(acrylamide-co-methacrylic acid), P(AAm-co-MAAc)) has temperature-regulable mechanical properties (its stiffness increases significantly at low temperatures, facilitating demolding; it regains softness and stretchability at room temperature) and shape memory characteristics. The dynamic regulation of wettability (contact angle) and adhesion performance can be achieved by adjusting the tilt angle of the microcolumns Figure 4a,b.

Micropillar array technology can also be combined with fluorescent labeling or image analysis techniques to monitor dynamic biological processes such as cell proliferation, migration, and mechanical responses in real time. This combination not only improves the richness and accuracy of the data, but also provides a new perspective for studying the mechanical interactions between cells and the microenvironment [87]. This study proposes the use of single-cell osmotic swelling dynamics as a physical biomarker. Through a microfluidic device, single cells are captured and subjected to hypotonic stimulation, with real-time monitoring of swelling dynamic parameters (maximum volume change, time to reach maximum volume, and swelling rate). This enables high-precision classification of different cell types (such as HeLa, HEK, Jurkat, and A20) with an accuracy rate of 99%, and can also detect cytoskeletal disturbances Figure 4c,d. The working principle of piezoelectric sensors depend on the change in crystal structure within the material. When the external mechanical pressure exerted by the outside surpasses a certain threshold, the internal electric dipole inside the material will be displaced, resulting in charge separation on the surface of the material, resulting in the generation of a voltage signal. The intensity of this signal is directly proportional to the applied force, allowing for the inference of force magnitude through voltage measurement. This characteristic has led to a wide range of applications in medical, industrial, and consumer electronics [88]. Conventional force sensors face many challenges when tasked with monitoring complex three-dimensional soft tissues, primarily due to the unique structural and mechanical properties of myocardial organoids. Therefore, researchers have developed a soft resistance force sensor based on an ultrasensitive nano-cracked platinum membrane, which is able to make good contact with organoids, thereby enabling real-time, wireless monitoring of the contractility of myocardial organoids [89]. A flexible force-sensing membrane based on a nano-cracked platinum film was developed and integrated into fully soft culture wells [90]. Through an “AFM-like” soft contact process, it establishes reliable contact with cardiac organoids, enabling real-time detection of the organoids’ contractile force and beating patterns (such as dynamic changes in electrical stimulation, drug treatment, and disease modeling), and supports wireless data transmission (realized through a Bluetooth module for remote monitoring), as shown in Figure 4e–g.

The integration of multimodal sensing systems has revolutionized the biomedical field, especially in the simultaneous monitoring of mechanical and biochemical signals. Real-time data derived from electrophysiological signals can be combined with optical imaging modalities, such as fluorescence imaging, to simultaneously capture both the electrical activity and morphological changes of cells. The advantage of this approach is that it provides more comprehensive biological information, enabling researchers to analyze biological phenomena at higher spatial and temporal resolution [88,89,90]. The application of this integrated system to monitor the electrophysiological responses and morphological changes of cardiomyocytes under different mechanical loads provides important data that supports for the study of cardiac diseases [91]. Furthermore, the combination of flexible electronics and microfluidic technology has opened up a new direction for the development of biosensors. This combination facilitates the execution of more complex tasks within a compact framework, especially in applications that require real-time monitoring and analysis of biological signals [92]. In the future, the combination of flexible electronic devices and microfluidic technology will provide more opportunities for innovation in medical and biological science research [93]. At the same time, interdisciplinary collaboration will be key to advancing the development of this field, especially the cross-disciplinary integration of materials science, engineering and biomedicine, which will help to promote the advancement and application of mechanical force sensor technologies.

### 2.3. Sensors That Detect Organoid Marker Signaling Molecules

#### 2.3.1. Optical Biosensors

Biosensors can be classified according to their signal conversion principle and the nature of the biomolecular components utilized. The primary classifications include electrochemical sensors, optical sensors, and piezoelectric sensors, among others. Notably, optical sensors have attracted great attention in recent years due to their high sensitivity and non-destructive detection advantages. Fluorescence sensors use changes in fluorescence signals to detect the presence of target molecules [94,95], while Raman spectroscopy sensors use molecular vibration information for highly selective detection. As research progresses, there has been a trend towards the convergence of spectroscopic techniques, exemplified by the integration of fluorescence and Raman spectroscopy, which can provide richer chemical information and specificity molecular identification [96].

**Figure 5:**
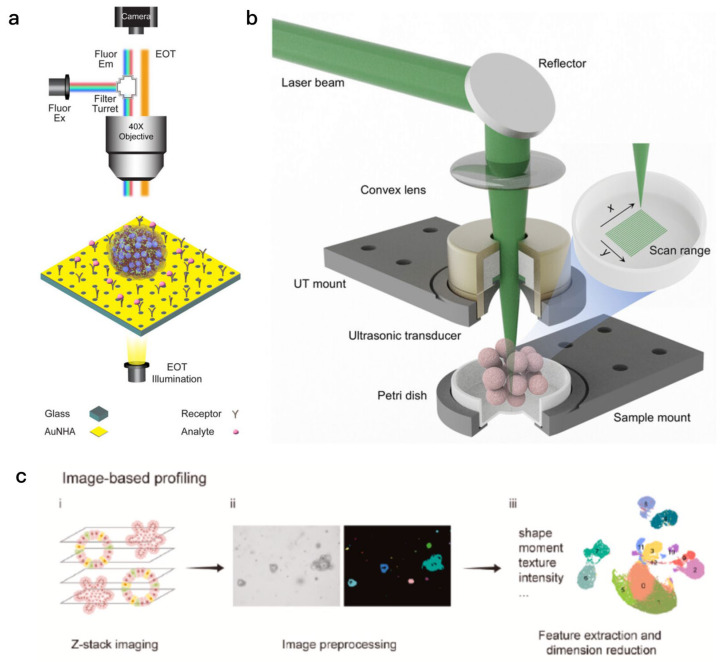
(**a**) The optical system enables simultaneous label-free plasmonic and fluorescence imaging of single spheroids: AuNHA chip functionalized, transmission mode for monitoring extra-spheroid activities, reflection mode for intra-spheroid monitoring, with sCMOS camera, etc. for image acquisition [97]. (**b**) Schematic of the LFOPI system for 3D photoacoustic imaging of tumor organoids [99]. (**c**) Image-based analysis workflow: cultured CRC organoids undergo z-stack imaging, 401 phenotypic features extracted after preprocessing, and dimensionally reduced to UMAP visualization [98].

Fluorescence imaging techniques have been widely used in organoid research, especially in detecting tumor cell migration [97] and the responses of tumors to pharmacological agents [98,99]. Based on this, a multimodal imaging platform integrating label-free nanoplasmonic biosensing and fluorescence microscopy was developed to simultaneously monitor the internal and external dynamics of single spheres (such as tumor spheroids) (Figure 5a). A label-free organoid photoacoustic imaging (LFOPI) system enables high-resolution 3D imaging and volume analysis of tumor organoids for drug screening (Figure 5b). Additionally, an image-based analysis workflow combined with deep learning technology was developed to analyze the morphological heterogeneity of colorectal cancer organoids and their correlation with functional parameters (such as viability and apoptosis) (Figure 5c). Förster resonance energy transfer (FRET)-based biosensors are versatile tools for obtaining insights into various biological processes. Their working principles are based on nonradiative energy transfer from donor to acceptor fluorophores. This energy transfer is responsible for a change in fluorescence intensity, which provides a basis for the detection of biomolecules. Advantageous features of FRET biosensors include their high sensitivity and specificity [100]. Fluorescent calcium imaging enables live-cell calcium imaging in three-dimensional myocardial organs, which provides an important tool for studying physiological processes related to cardiac function [101]. This technique not only enables monitoring changes in intracellular calcium concentration at the cellular level, but also provides a new perspective on how tumor cells affect the surrounding microenvironment [102]. Raman spectroscopy is a spectroscopic analysis method based on molecular vibrational scattering, which can obtain molecular information by analyzing the frequency change of the scattered light after the incident light interacts with the sample. By monitoring these frequency changes, Raman spectroscopy can generate characteristic molecular fingerprints that can be used to monitor the dynamics of biological samples to help study disease progression or efficacy assessment [18]. This includes the analysis of biomolecules such as DNA, RNA, and proteins, which help us to better understand disease mechanisms and biological processes [103]. By optimizing the Raman imaging system, scientists have developed a high-throughput linear illumination Raman microscope, which can acquire a large amount of Raman spectral data in a short period of time, so as to achieve rapid imaging and dynamic monitoring of organoids [104]. In studies of Alzheimer’s disease, Raman spectroscopy was able to accurately classify different patients and their plasma samples [105].

Surface-enhanced Raman spectroscopy (SERS) is a technique that uses the surface plasmon resonance effect of metal nanostructures to significantly enhance the Raman scattering signal, providing unmatched sensitivity for detecting molecules at low concentrations [106]. Trace amounts of metabolites in organoid cells were successfully detected by SERS technology, using functionalized gold nanoparticles as SERS substrates, which demonstrated superior sensitivity and specificity [107]. SERS not only improves detection sensitivity, but also provides molecular characterization information for molecular identification and analysis of complex biological samples. SERS can also enable real-time monitoring of metabolites in organoids, which provides the possibility of early diagnosis and personalized treatment of various diseases [108].

In the research focused on the combination of biosensors and organoids, the integration and miniaturization of the system are the key to realize their practical application. The integration of optical sensors and organoid culture systems enables real-time monitoring and analysis of biological samples, which is essential for understanding the interactions between cells and their microenvironment [109]. The use of miniaturized optical sensors in combination with organoid models enables efficient detection of small samples, which provides a new solution for rapid detection in resource-constrained environments [47]. Fluorescence spectroscopy is known for its high sensitivity and is particularly suitable for detecting biomolecules at low concentrations. However, in some cases, fluorescence background signals in biological samples may interfere with results, especially when detecting complex samples [110]. To address this issue, Raman spectroscopy, as a technique that does not require sample pretreatment, can provide information about the chemical structure of molecules, and therefore can be used in combination with fluorescence spectroscopy to complement their respective shortcomings [111,112]. When Raman spectroscopy is combined with fluorescence spectroscopy, the chemical composition and concentration information of molecules can be obtained at the same time, which greatly improves the comprehensiveness and accuracy of the analysis. In this review, we compare the advantages and disadvantages of Fluorescence Imaging, Raman Spectroscopy and Fluorescence–Raman Hybrid Technology (Table 2).

#### 2.3.2. Label-Free Sensing

Label-free technology refers to a technology that can directly detect changes in the physical or chemical properties of the target molecule without the need for an exogenous label. By directly detecting the changes in the physical or chemical properties of organoids, the interference of exogenous markers is avoided, and the reliability of the experimental results is improved. According to different detection mechanisms, label-free technologies can be divided into several categories, which mainly include electrochemical sensing technology, optical sensing technology, and mass spectrometry imaging technology. Electrochemical sensors, such as impedance sensors, are able to identify target molecules by monitoring changes in current or voltage [113]. For example, impedance sensors can monitor the electrophysiological properties of cells in real time, reflecting changes in their growth state and function [1]. Optical sensing techniques, especially surface plasmon resonance (SPR), use the interactions of light with surface plasma to monitor changes in the refractive index caused by the binding of biomolecules, which are characterized by high sensitivity and real-time detection [114]. In tumor organoid studies, SPR has been used to analyze the effects of different drugs on tumor cells and to help researchers to evaluate the efficacy and safety of drugs [115]. Mass spectrometry imaging (MSI) provides researchers with a label-free metabolomics analysis tool that enables the probing of metabolic changes in organoids at the cellular level. The application of this technique in research liver organoids has shown its importance in drug metabolism and toxicity assessment [116]. It can also provide spatial information on the distribution of molecules by analyzing changes in molecular mass in samples, which is widely used in tumor analysis and drug distribution studies [117].

The core principle of impedance sensors is to reflect the physical or chemical properties of substances based on their opposition to alternating current (impedance). Specifically, a sensor typically includes one or more pairs of electrodes, which are placed around or inside the sample to be measured (e.g., organoids). When a weak alternating current of a specific frequency is applied across the electrodes, the conduction path of the current is affected by the electrical properties of the sample (such as conductivity and dielectric constant), resulting in different impedance values (impedance is a complex quantity combining resistance, inductance, and capacitance, whose magnitude is frequency-dependent). Organoids are three-dimensional cell aggregates formed by the differentiation of stem cells or organ precursor cells, with structures and functions similar to real organs. Their physiological states (e.g., cell viability, proliferation, morphology, and barrier function) are closely related to electrical properties. Impedance sensors detect these parameters by capturing these correlations. The cell membrane of living cells has an intact lipid bilayer, which strongly opposes current (high impedance). When cells die, the membrane ruptures, releasing intracellular substances and increasing conductivity (low impedance). Thus, a decrease in impedance reflects reduced cell viability in organoids. During organoid proliferation, the increased number of cells and tighter intercellular connections block the current conduction path, leading to a rise in impedance. The proliferation rate of organoids can be quantified by monitoring the time-dependent increase in impedance [118]. Changes in organoid morphology (e.g., size, compactness, polarity) affect the contact area between cells and electrodes, as well as the current conduction path. For example, dispersion or disintegration of organoids causes a significant drop in impedance, while a more compact structure results in higher impedance [119]. Certain organoids (e.g., intestinal or brain organoids) have barrier structures similar to epithelia or endothelia, whose integrity can be measured by transepithelial electrical resistance (TEER). Intact barriers with tight junctions prevent ion flow, resulting in high TEER (high impedance); barrier damage (e.g., due to inflammation or toxin exposure) leads to decreased TEER, which can be monitored in real time by impedance sensors. In summary, by continuously monitoring dynamic impedance changes of organoids under different physiological states, impedance sensors enable real-time, non-invasive detection of multiple physiological parameters without the need for labeling, providing an efficient quantitative tool for organoid development research and drug screening.

In SPR sensors, a thin metal film such as gold or silver is often used as the sensing surface. When the molecule to be measured binds to the metal surface, it causes a change in the refractive index of the surface, which causes a shift in the SPR angle. This shift can be monitored in real time by an optical detection system to obtain kinetic information on molecular interactions. The high sensitivity of this process enables the label-free detection of biomolecules, drugs, and other chemicals by SPR technology with the advantages of rapid, real-time, and high-throughput [120]. SPR sensors can monitor the binding process of drugs to target proteins in real time, thus providing important data support for new drug development [121,122].

Mass spectrometry imaging (MSI) operates on the principle that a sample (such as an organoid) is first appropriately processed (e.g., thin sections are prepared to preserve spatial information of its three-dimensional structure), followed by the conversion of molecules (including metabolites, proteins, lipids, small-molecule drugs, etc.) on the sample surface into charged ions using specific ionization techniques (such as matrix-assisted laser desorption/ionization, MALDI, or secondary ion mass spectrometry, SIMS). These ions are separated in a mass spectrometer and detected based on their mass-to-charge ratio (m/z), while the instrument precisely records the spatial coordinates where each ion is generated (corresponding to specific positions on the sample surface). By integrating the mass-to-charge ratio information, signal intensity, and spatial positions of all detected ions, data analysis software can ultimately reconstruct distribution images of specific molecules on the sample surface, directly linking molecular composition to spatial location [123]. In the detection of organoid physiological parameters, this technology exhibits unique advantages: as three-dimensional cell aggregates that mimic the structure and function of in vivo organs, organoids’ physiological states (such as cellular heterogeneity, metabolic activity, expression and distribution of specific biomolecules, and local responses to drugs) are closely related to the spatial distribution of molecules. MSI can detect spatial differences of multiple molecules without damaging the integrity of organoids—for instance, analyzing the distribution of energy metabolites (such as ATP and lactic acid) can reflect cellular activity in different regions; detecting the spatial enrichment of intercellular signaling molecules (such as cytokines) can identify functionally specialized regions; and tracking the distribution of drugs and their metabolites can evaluate the local response efficiency of organoids to drugs, thereby providing comprehensive, in situ, multi-molecular level spatial information for analyzing organoid physiological parameters [124].

**Table 2:** Optical biosensors of key technical principles, advantages and disadvantages.

Category	Principle	Advantages	Disadvantages
Fluorescence Imaging	Detects target molecules or biological processes by monitoring changes in fluorescence signals (e.g., intensity, wavelength shifts) [94,95].	1. High sensitivity, suitable for low-concentration biomolecule detection. 2. Non-destructive, enabling live-cell and organoid monitoring. 3. Widely applied in studying tumor migration, drug responses, and intracellular dynamics (e.g., calcium imaging in cardiac organoids) [97,98,99,100,101,102].	1. Susceptible to fluorescence background interference in complex biological samples [110]. 2. May require labeling, which could affect biological activity.
Raman Spectroscopy	Obtains molecular “fingerprints” by analyzing frequency shifts of scattered light after interaction with molecules, based on molecular vibrational scattering [18].	1. Label-free, no need for sample pretreatment. 2. Provides detailed chemical structure information (e.g., DNA, RNA, proteins) [123]. 3. Enables high-throughput analysis with optimized systems (e.g., linear illumination Raman microscope) [104]. 4. Applicable for disease classification (e.g., Alzheimer’s disease) [105].	1. Lower sensitivity compared to fluorescence spectroscopy for ultra-low concentration analytes. 2. Requires longer acquisition times for high-quality data.
Fluorescence-Raman Hybrid Technology	Integrates fluorescence and Raman spectroscopy to combine their signal outputs [96].	1. Complements respective shortcomings: fluorescence provides high sensitivity, while Raman offers chemical structure specificity. 2. Enables simultaneous acquisition of molecular concentration and chemical composition, improving analysis comprehensiveness and accuracy [111,112].	1. Increased system complexity due to integration of two techniques. 2. Potential signal crosstalk between fluorescence and Raman signals, requiring careful optimization.

Label-free sensing technologies, which enable direct detection of changes in the physical or chemical properties of target molecules without exogenous labels, avoid interference from external markers and improve the reliability of experimental results, with key technologies including electrochemical sensing, optical sensing, and mass spectrometry imaging. Impedance sensors, a type of electrochemical sensor, identify target molecules or cell states by monitoring changes in electrical impedance caused by cell growth, adhesion, or barrier function, allowing real-time monitoring of electrophysiological properties, evaluation of multiple biological characteristics like barrier function and proliferation, and cell classification based on frequency-dependent responses, though they provide less specific molecular information and are affected by environmental factors. Surface plasmon resonance (SPR), an optical sensing technique, uses light interactions with surface plasmons on thin metal films to monitor refractive index changes from biomolecular binding, enabling label-free, high-sensitivity, real-time detection of molecular interactions, supporting high-throughput analysis and drug efficacy evaluation, but it relies on the metal film surface and is sensitive to non-specific binding. Mass spectrometry imaging (MSI) combines mass spectrometry with imaging to simultaneously monitor the spatial distribution of multiple molecules in biological samples by ionizing molecules on organoid surfaces, analyzing their mass-to-charge ratios, and generating spatial images, providing label-free metabolomics analysis at the cellular level and useful for drug metabolism studies, though it requires appropriate matrix selection for organoid sections, increasing sample preparation complexity, and may have lower spatial resolution compared to optical imaging for small-scale structures. These technologies collectively offer diverse approaches to study organoid physiology, drug responses, and disease mechanisms. In this review, we compare the advantages and disadvantages of impedance sensors, surface plasmon resonance (SPR) and mass spectrometry imaging (MSI) (Table 3).

Section 2 systematically reviews the key biosensing technologies integrated with organoids to monitor their microenvironment, electrophysiological properties, and signaling molecules. For microenvironment monitoring, microfluidic systems simulate physiological conditions and integrate sensors for real-time tracking of parameters like pH and nutrient levels; electrochemical sensors (enhanced by nanomaterials and molecularly imprinted polymers) detect metabolites with high sensitivity; and field-effect transistors (FETs) enable rapid, label-free monitoring of ions and metabolites. In electrophysiological monitoring, microelectrode arrays (MEAs)—including 3D configurations—record neural and cardiac electrical activity, while mechanical force sensors quantify contractility and biomechanical responses. For signaling molecule detection, optical sensors (fluorescence, Raman spectroscopy, and SERS) provide high-resolution molecular and structural insights, complemented by label-free technologies (impedance sensors, SPR, MSI) that avoid exogenous markers, enhancing result reliability. In this review, we compare the advantages and disadvantages of these technologies (Table 4). Collectively, these technologies enable multi-dimensional, real-time analysis of organoids, overcoming limitations of single-modality sensing and laying the groundwork for advanced applications in biomedicine.

## 3. Integration of Multimodal Sensors in Organoids

The integration of multimodal sensors represents a pivotal advancement in organoid research, enabling the simultaneous capture of complementary biological signals to overcome the limitations of single-modality sensing. By strategically combining technologies such as microfluidics, electrochemical sensors, microelectrode arrays (MEAs), optical imaging, and mechanical force transducers on a unified platform, researchers can monitor multiple parameters—including metabolic dynamics, electrophysiological activity, structural changes, and mechanical responses—of organoids in real time, thereby obtaining comprehensive biological information that single sensors cannot provide.

Specific implementation strategies for such integration are multifaceted. Hardware-wise, microfluidic chips often serve as the core framework, facilitating the co-localization of diverse sensors. For instance, a microfluidic system can integrate optical modules (e.g., fluorescence microscopy and Raman spectroscopy) to visualize molecular distributions and structural changes [18], electrochemical sensors (e.g., microelectrodes or FETs) to detect metabolites like glucose and lactate, and mechanical sensors (e.g., micropillar arrays or piezoelectric devices) to measure contractile forces or cell adhesion. This physical integration ensures that all sensors probe the same organoid or region, allowing direct correlation between, for example, calcium flux (via fluorescence) and electrical activity (via MEA) in cardiac organoids. In software and data terms, synchronization is critical: shared hardware triggers (e.g., clock signals) ensure simultaneous data acquisition across modalities, while machine learning algorithms (such as support vector machines and convolutional neural networks) enable the fusion of heterogeneous data—for example, merging Raman spectral data (molecular composition) with impedance measurements (cell viability) to classify organoid phenotypes with high accuracy [60,125]. Additionally, modular designs allow flexible swapping of sensor components, adapting to specific research needs [126] (e.g., combining MEA and calcium imaging for neural organoids, or SERS and impedance sensing for tumor organoids).

The advantages of such multimodal systems are substantial. Firstly, they enhance biological insight by capturing interdependent physiological processes. For example, in neural organoids, combining MEA (electrical activity) with fluorescence imaging (calcium dynamics) and Raman spectroscopy (neurotransmitter levels) reveals how synaptic firing correlates with intracellular signaling and metabolic shifts during drug exposure. In tumor organoids, pairing surface plasmon resonance (SPR) (drug–target binding) with mass spectrometry imaging (MSI) (drug distribution) and mechanical sensing (invasion force) clarifies how drug efficacy relates to spatial penetration and biomechanical changes. Secondly, cross-validation between modalities improves data reliability: impedance measurements indicating increased cell proliferation can be validated by fluorescence imaging of proliferation markers (e.g., Ki67), while discrepancies (e.g., impedance changes unaccompanied by marker expression) may signal artifacts like matrix stiffness alterations. Thirdly, integrated systems streamline workflows, reducing sample handling and enabling high-throughput screening—for instance, a multimodal organoid-on-a-chip can simultaneously assess drug-induced changes in electrophysiology (via MEA), metabolism (via electrochemical sensors), and morphology (via optical imaging), accelerating applications in personalized medicine [127,128].

Moreover, the integration of AI-driven data analysis with multimodal sensors further amplifies their utility. By processing data from biosensors and imaging techniques through chemical imaging and machine learning, researchers can real-time monitor biological reactions and drug effects, adapting treatment strategies to individual organoid responses [129]. This holistic approach not only improves experimental efficiency and reproducibility but also provides a powerful tool for decoding complex biological mechanisms, advancing drug screening, and facilitating precision medicine.

## 4. Challenges and Prospects of Organoids and Biosensors

### 4.1. Challenge

In the integration of biosensors with organoids, several critical challenges persist, requiring nuanced elaboration to clarify their impact and underlying complexities:

The sensitivity of biosensors remains a primary bottleneck, particularly in detecting low-abundance biomarkers (e.g., cytokines, metabolites) at picomolar or lower concentrations—levels critical for early disease detection and subtle physiological changes in organoids [130]. This limitation stems from inherent signal-to-noise ratios in complex biological matrices (e.g., culture media with proteins, cellular debris), where non-specific binding and background interference mask target signals. For instance, electrochemical sensors often suffer from fouling by biomolecules adhering to electrode surfaces, gradually reducing response accuracy over time [131].

Stability is further compromised by dynamic environmental factors in organoid cultures. Fluctuations in temperature (35–39 °C) can alter the conductivity of nanomaterial-based sensors; variations in pH (e.g., 6.8–7.6 in hypoxic tumor organoids) affect the reactivity of enzyme-based electrochemical sensors; and cumulative changes in cellular metabolites (e.g., lactate, reactive oxygen species) can degrade sensor materials (e.g., oxidation of graphene or gold nanostructures) [131]. These factors collectively lead to drift in sensor output, undermining the reliability of long-term monitoring (e.g., 7–30 days of organoid culture), a critical requirement for studying developmental processes or chronic drug responses.

Organoid heterogeneity—encompassing genetic, morphological, and functional variability—poses substantial challenges for consistent biosensor readouts. Genetically, organoids derived from different donors (or even different biopsy sites of the same donor) exhibit divergent gene expression profiles, e.g., varying drug transporter levels in colorectal cancer organoids, which directly affect drug response patterns detected by biosensors [132]. Morphologically, even isogenic organoids often display differences in size (50–500 μm), cell type composition (e.g., epithelial-stromal ratios), and matrix integration, leading to uneven sensor-organoid contact (e.g., inconsistent electrode coupling in MEA systems) and skewed signal acquisition [133].

This heterogeneity complicates the standardization of sensing protocols. For example, impedance sensors monitoring barrier function may yield conflicting results for intestinal organoids from the same donor batch due to variable tight junction formation, making it difficult to establish baseline thresholds for “normal” vs. “diseased” states. Addressing this requires not only improved organoid culture techniques (e.g., uniform scaffold matrices) but also sensor designs that account for spatial variability—an unmet need in current technologies.

Biocompatibility mismatches between sensors and organoid microenvironments Achieving long-term biocompatibility remains elusive, as sensor materials often disrupt organoid physiology or are themselves degraded by the biological microenvironment. Short-term compatibility (e.g., 1–7 days) is relatively manageable with inert polymers (e.g., PDMS) or biocompatible metals (e.g., gold), but extended cultures (≥ 14 days) expose critical issues: Material-cell interactions: Rigid sensor substrates (e.g., silicon) can alter organoid mechanics, triggering abnormal YAP/TAZ signaling and disrupting differentiation—particularly problematic for mechanosensitive organoids like cardiac or neural spheroids [134]. Leachables and degradation: Some sensor coatings (e.g., polymeric encapsulants) release monomers or additives that induce cytotoxicity; conversely, organoid-secreted proteases (e.g., matrix metalloproteinases) can degrade sensor surfaces, impairing signal transduction [135]. Immunogenicity: Animal-derived materials (e.g., Matrigel) used in organoid cultures, though supportive of growth, introduce batch-to-batch variability and potential immune reactions when combined with sensor components, complicating translation to clinical-grade systems [136].

Synthetic alternatives (e.g., hydrogels with defined peptide motifs) aim to address these issues but often lack the bioactivity to support organoid maturation, creating a trade-off between biocompatibility and functional relevance [137,138].

These challenges are interconnected: sensor sensitivity issues exacerbate the impact of organoid heterogeneity, while biocompatibility problems limit the stability of long-term sensing. Resolving them will require interdisciplinary advances—from materials engineering (e.g., self-cleaning sensor surfaces) to organoid standardization (e.g., AI-driven culture optimization)—to realize the full potential of organoid-biosensor systems.

### 4.2. Future Prospects

The development of new biosensors has shown great potential in organoid monitoring, especially in the application of nanomaterials and flexible electronics. Due to their unique physical and chemical properties, nanomaterials can improve the sensitivity and selectivity of sensors. For example, carbon-based nanomaterials, such as graphene and carbon nanotubes, are widely used in biosensors [139]. These materials have good conductivity and biocompatibility, making them ideal for real-time monitoring of biomarkers [18]. In addition, advances in flexible electronics have enabled sensors to be embedded into the three-dimensional structure of organoids, enabling non-invasive monitoring of dynamic changes in organisms. This flexibility not only improves the accuracy of surveillance, but also opens up new possibilities for personalized medicine, especially in the study of cancer and other complex diseases [108]. By combining these sensors with organoids, researchers can obtain real-time data on cellular behavior, leading to a better understanding of disease development mechanisms and their response to treatment [140].

With the rapid development of biosensors and organoid technology, the future of personalized medicine is becoming clearer. Organoids can be used to predict a patient’s responses to a specific drug by reflecting the genomic characteristics and microenvironment of the patient’s tumor, thereby enabling more precise treatment options [141]. Combining biosensors, especially those can monitor biomarkers in real time, can further improve the precision of personalized medicine. For example, biosensors can monitor biomarker changes in organoids during drug processing, so that doctors can assess the patient’s responses to the drug in real time and adjust the treatment regimen in a timely manner [142]. At the same time, the combination of organoids and biosensors can be used to rule out drug ineffectiveness at an early stage, thereby improving the screening efficiency and success rate [143].

The rapid commercialization of organoid-biosensor technologies has fostered a vibrant ecosystem of companies and start-ups, bridging academic innovation with practical applications in drug discovery, personalized medicine, and disease modeling, with these entities leveraging diverse sensing modalities—from microfluidics to optical and electrochemical systems—to advance the field; established players include Emulate Bio (USA), a pioneer in “Organs-on-Chips” technology that integrates microfluidic systems with optical and electrochemical sensors to mimic human organ functions, whose platforms like the Human Emulation System™ monitor organoid responses to drugs via real-time metabolite detection (e.g., glucose, lactate) and barrier function analysis, enabling pharmaceutical companies to assess efficacy and toxicity with high precision, Axion Biosystems (USA), which specializes in microelectrode array (MEA) technology, with their Maestro^®^ systems widely used for long-term electrophysiological monitoring of neural and cardiac organoids, combining high-throughput electrical signal recording with software for analyzing network dynamics to support neuroscience research and cardiotoxicity testing, and Organovo (USA), focused on 3D bioprinted organoids, which integrates label-free sensing technologies like mass spectrometry imaging (MSI) and surface plasmon resonance (SPR) to analyze organoid morphology and molecular profiles, with their ExThera^®^ liver organoids paired with biosensors enabling drug metabolism and toxicity assessment; emerging start-ups include Xellar Biosystems (USA, founded 2021), a trailblazer in “3D-Wet-AI” technology that combines organoid chips, 3D imaging, and AI to accelerate drug discovery, using high-content optical sensors and machine learning to analyze 3D cellular responses with applications in oncology and neurodegenerative diseases, and participating in the OASIS Consortium to collaborate with the FDA and major pharmaceutical companies on standardizing preclinical toxicity testing, Qingyuan Zhixin (Shenzhen) Biotechnology (China, founded 2024), which specializes in 3D-printed organoids integrated with AI-driven biosensing, with proprietary 3D bioprinters generating “micro-organs” from stem cells paired with electrochemical sensors for real-time metabolite monitoring, and an AI system enabling high-throughput analysis of drug responses targeting personalized cancer therapy, FinalSpark (Switzerland, founded 2024), focused on bioelectronic interfaces, whose Neuroplatform combines human brain organoids with multi-electrode arrays (MEAs) to create biocomputing systems, using microfluidics for nutrient perfusion and optical sensors for monitoring neural activity with potential applications in low-energy computing and neurodegenerative disease modeling, and LuoHua Bio (China, founded 2019), a leader in organ-on-a-chip systems that has developed over 10 chip models (e.g., lung, liver) integrated with impedance and optical sensors, with their tumor organoid biobank paired with AI analysis supporting high-throughput drug screening and personalized treatment design for cancers; these companies drive innovation by addressing scalability, standardization, and clinical translation—key challenges in organoid-biosensor integration—with their work accelerating the adoption of these technologies in pharmaceuticals and clinical settings, paving the way for more efficient drug development and precision medicine.

The combination of organoids and biosensors has opened up a new path for personalized medicine, making drug screening and disease treatment more accurate and personalized. With the continuous advancement of technology, more beds based on this combination may emerge in the future to promote the development of precision medicine.

## 5. Conclusions

With the continuous development of biosensors and organoid technologies, the precise interactions between the two opens up new opportunities for multimodal physiological parameter monitoring. This interaction not only greatly enriches the way physiological data is obtained, but also provides an important foundation for the early diagnosis of diseases, the evaluation of drug responses, and the implementation of personalized medicine. However, despite the abundance of research in this area, there are still many challenges that need to be addressed.

Firstly, the accuracy and sensitivity of biosensors are important factors affecting their practical applications. Existing sensors often face problems such as signal noise and interference when monitoring a variety of physiological parameters, which calls into question the reliability of the data. Therefore, how to improve the performance of the biosensors so that it can work stably in a complex physiological environment is one of the keys to future research.

Secondly, although the progress of organoid technology provides new possibilities for personalized medicine, how to effectively combine it with biosensors to form a complete monitoring system still needs to be further explored. The physiological characteristics and individual differences of organoids make standardization and reproducibility difficult. To achieve this, researchers need deeper interdisciplinary collaboration in model building, data integration, and more to ensure that the interaction between biosensors and organoids is a true reflection of physiological state.

In terms of disease modeling, the application potential of intelligent multimodal monitoring systems is huge. By monitoring different physiological parameters in real time, researchers can gain a more complete picture of the disease process and develop more precise intervention strategies. This not only contributes to the in-depth basic research, but also provides new ideas for clinical treatment. However, how to effectively integrate multimodal data and extract meaningful information is the key to achieve this goal.

Looking ahead, technological innovation will play an indispensable role in driving the development of intelligent multimodal monitoring systems. The application of new materials, the optimization of sensor design, and the improvement of data analysis methods will provide new possibilities for improving the performance of monitoring systems. In addition, interdisciplinary collaborations will bring new perspectives and approaches to the field. For example, the combination of computer science and biomedicine can provide strong support in data processing and analysis, and promote the development of intelligent monitoring systems to a higher level.

In summary, the precise interactions between biosensors and organoids provides unprecedented opportunities for multimodal physiological parameter monitoring, but it also faces many challenges. In the future, through technological innovation and interdisciplinary cooperation, intelligent multimodal monitoring systems are expected to play an important role in disease modeling, drug development, and personalized medicine. Only based on continuous optimization of technology and strengthened cooperation can we better achieve this goal and promote the progress and development of medical research.

## Figures and Tables

**Table 1:** Electrochemical sensors of key technical principles, advantages and disadvantages.

Category	Principle	Advantages	Disadvantages
Electrochemical sensors (basic)	Generate electrical signals (current, potential changes) through the reaction between biological recognition elements on the electrode surface and target substances to reflect the concentration of the substances.	High sensitivity, fast response speed, can be miniaturized and integrated, suitable for real-time monitoring.	Face challenges in sensitivity and selectivity in complex biological environments (such as organoid culture media) and are susceptible to interfering substances.
Material-modified electrochemical sensors	Modify electrodes with high specific surface area nanomaterials (such as carbon nanotubes, graphene, MXene and other two-dimensional materials) to enhance electrochemical reaction rates; optimize chemical properties through surface functionalization/modification of electrodes to improve specificity for target molecules.	1. Nanomaterials significantly enhance the electrochemical reaction rate of electrodes and improve the response capability of sensors. 2. Surface modification can reduce interference and enhance specificity for target molecules. 3. New two-dimensional materials (e.g., MXene) exhibit excellent electrochemical performance and sensitivity, suitable for detection in complex biological environments.	Rely on sophisticated material modification techniques; the dispersibility and stability of nanomaterials may affect the performance consistency of sensors.
Molecularly Imprinted Polymer (MIP) technology	Construct specific recognition sites on the electrode surface to accurately identify target analytes.	1. Significantly improve the selectivity of sensors for target molecules and reduce interference from structurally similar compounds (e.g., glucose sensors eliminate interference from other sugars). 2. Relatively low preparation cost and good stability.	The uniformity of recognition sites may be insufficient, and non-specific binding may occur in complex biological samples.
Organic Electrochemical Transistors (OECTs)	Act as signal amplifiers, amplifying detection signals through the sensitivity of organic semiconductor channels to changes in ion concentration.	1. Greatly improve sensor sensitivity, enabling the detection of extremely low concentrations of biomarkers. 2. Suitable for integrated design, enhancing real-time monitoring capabilities.	Sensitive to environmental conditions (such as humidity and temperature); long-term stability needs further optimization.
Integration and miniaturization of electrochemical sensors	Rely on precision manufacturing technologies such as 3D printing and microfabrication to miniaturize sensors and integrate them into the same system (e.g., organoid culture systems) for simultaneous multi-parameter monitoring.	1. Reduce costs, improve portability and real-time monitoring capabilities. 2. Can be directly embedded into culture systems to continuously monitor metabolic indicators (e.g., glucose, lactate), enhancing monitoring accuracy. 3. Streamline experimental procedures, reduce sample handling, and minimize potential interference.	Miniaturized manufacturing requires high process precision; the compatibility and long-term stability of integrated systems need continuous optimization.

**Table 3:** Label-free sensing of key technical principles, advantages and disadvantages.

Category	Principle	Advantages	Disadvantages
Impedance Sensors	Identify target molecules or cell states by monitoring changes in electrical impedance (current/voltage fluctuations) caused by cell growth, adhesion, or barrier function variations [1,113].	1. Enables real-time monitoring of electrophysiological properties, reflecting cell growth, function, and viability [1,112]. 2. Evaluates multiple biological characteristics (barrier function, cell adhesion, proliferation) [118]. 3. Frequency-dependent responses allow cell classification and state monitoring [119]. 4. Label-free, avoiding interference from exogenous markers.	1. Limited to monitoring changes related to cell quantity or structure (e.g., junctions), providing less specific molecular information. 2. May be affected by environmental factors (e.g., medium conductivity) leading to signal variations.
Surface Plasmon Resonance (SPR)	Uses interactions between light and surface plasmons (on a thin metal film, e.g., gold/silver) to monitor refractive index changes caused by biomolecular binding on the surface, detected via shifts in SPR angle [114,120].	1. Label-free detection based on refractive index changes, suitable for real-time monitoring of biomolecular interactions [114]. 2. High sensitivity and rapid response, enabling kinetic analysis of molecular binding [119]. 3. Applicable for drug efficacy evaluation and new drug development [115,121,122]. 4. Supports high-throughput analysis.	1. Relies on a metal film surface, limiting detection to molecules that can bind to the surface (requires specific immobilization). 2. Sensitive to non-specific binding, which may interfere with results in complex biological samples.
Mass Spectrometry Imaging (MSI)	Combines mass spectrometry with imaging: ionizes molecules on organoid surfaces using specific matrices, analyzes their mass-to-charge ratios via mass spectrometry, and generates spatial distribution images of molecular species [123,124].	1. Provides label-free metabolomics analysis with spatial distribution of multiple molecules in organoids [116,117]. 2. Enables cellular-level probing of metabolic changes, useful for drug metabolism and toxicity assessment [116]. 3. Combines mass spectrometry with imaging to offer both molecular identity and spatial location [123].	1. Requires appropriate matrix selection for organoid sections to enhance ionization efficiency [124], adding complexity to sample preparation. 2. May have lower spatial resolution compared to optical imaging techniques for small-scale organoid structures.

**Table 4:** Comparison of various techniques applied in organoid-based biosensors.

Technical Type	Application in Organoids	Advantages	Disadvantages
Microfluidic Technology	1. Centered on microfluidic chips, realizing three-dimensional (3D) culture of organoids to simulate human organ functions. 2. Capable of mimicking tumor microenvironments and metastasis processes for drug testing and mechanism research. 3. Enabling efficient screening of drug compounds, such as anti-cancer drugs. 4. Iintegrating sensors to real-time monitor key parameters in culture media, including pH value, oxygen partial pressure, and nutrient concentration.	1. Can precisely control microenvironments, such as fluid perfusion and chemical gradients, promoting more biomimetic organ structures and functional maturation. 2. Can integrate sensors for real-time monitoring of cellular responses. 3. In drug screening, it can simulate in vivo drug metabolism processes to more accurately predict drug biological effects and toxicity. 4. Can address the problem of insufficient cell quantity in traditional culture.	1. Traditional microfluidic chips hardly replicate in vivo flow. 2. There are challenges in achieving in vivo functional vascularization of induced pluripotent stem cell (iPSC)-derived organoids in vitro. 3. Constructing vascularized organ chips remains difficult.
Electrochemical Sensors	1. Detecting biomarkers in organoids, such as metabolic molecules like glucose and lactate, to real-timely reflect the functional status of organoids. 2. Improving the detection capability for specific biomarkers through optimized design and adoption of new materials.	1. Based on the principle of electrochemical reactions, they can directly detect the concentration of target molecules. 2. The application of new two-dimensional materials, etc., enhances electrochemical performance and sensitivity. 3. The trend of miniaturization and integration reduces costs and improves portability and real-time monitoring capabilities.	1. Face challenges in sensitivity and selectivity in complex biological environments. 2. Are susceptible to environmental factors and culture materials, and sensor performance may fluctuate, affecting data reliability.
Microelectrode array (MEA) technology	1. Record or stimulate neural electrical activity, such as recording the spontaneous electrical activity of neural organoids to reflect the connections and network activities among neurons. 2. Record the electrophysiological characteristics of cardiac organoids for drug screening and regenerative medicine research. 3. Combine multimodal data fusion strategies with mechanical stress loading, etc., to quantify changes in electrical activity.	1. It can detect the electrical signals of neurons or cardiomyocytes in a high-throughput manner. 2. The three-dimensional electrode structure can increase the contact area with the organoids, improving the signal quality and stability. 3. And it can achieve long-term stable recording and precise regulation of the electrophysiological activities of the organoids.	1. Traditional planar electrodes are limited by space when contacting three-dimensional tissues, resulting in poor signal acquisition. 2. Some existing electrophysiological recording techniques cannot perform long-term suspension recording while maintaining the morphology of organoids.
Mechanical Force Sensors	1. Monitoring the response of organoids to external mechanical stimuli, such as the contractile force of cardiac organoids. 2. Combining with other technologies to synchronously monitor mechanical and biochemical signals, etc. 3. Valuating biomechanical properties of cells, such as adhesiveness and migration ability.	1. Can provide real-time and continuous biophysical data, revealing the interaction between cells and the microenvironment. 2. Emerging ultrasensitive sensors can achieve monitoring of tiny forces. 3. Integrating with multimodal sensing systems provides more comprehensive biological information.	1. Traditional force sensors face challenges in monitoring complex 3D soft tissues, and their contact and adaptability with organoids need improvement. 2. Some sensors may have stability issues during long-term use.
Optical Sensors	1. Optical sensors using fluorescence imaging to detect tumor cell migration and response to drugs, achieving live-cell calcium imaging of cardiac organoids. 2. Analyzing the chemical structure and metabolic changes of molecules in organoids through Raman spectroscopy. 3. Detecting low-concentration molecules by Surface-Enhanced Raman Spectroscopy (SERS) technology.	1. Feature high sensitivity and non-destructive detection advantages. 2. Can provide information on the chemical composition and concentration of molecules. 3. SERS technology has extremely high sensitivity in detecting low-concentration molecules. 4. Enables rapid imaging and dynamic monitoring of organoids.	1. Fluorescence sensors may be interfered by fluorescent background signals in biological samples. 2. Raman spectroscopy signals may be weak when detecting complex samples. 3. System integration and miniaturization still need further improvement.
Label-Free Sensing	1. Monitoring the electrophysiological properties, growth status, and barrier function, etc., of organoids through impedance sensors. 2. Analyzing the effect of drugs on tumor organoids using Surface Plasmon Resonance (SPR) technology. 3. Detecting metabolic changes in organoids by adopting Mass Spectrometry Imaging (MSI) technology.	1. No exogenous markers are required, avoiding interference from exogenous markers and improving the reliability of experimental results. 2. Impedance sensors can real-timely monitor multiple biological characteristics. 3. SPR technology has the characteristics of high sensitivity and real-time detection. 4. MSI can simultaneously monitor the spatial distribution of multiple molecules.	1. In cell classification and status monitoring by impedance technology, the impedance response differences of different cells or states need precise identification. 2. SPR technology has strong dependence on the sensing surface. 3. MSI technology has high requirements for sample processing and matrix selection, etc.
Nanomaterial Technology	1. Modifying electrodes to reduce interface impedance and enhance the electrochemical performance of electrodes, such as graphene-modified electrodes; serving as SERS substrates, such as gold nanoparticles, to enhance Raman scattering signals. 2. Being used to develop new biosensors, such as sensors made of carbon-based nanomaterials.	1. Nanomaterials have unique physical and chemical properties, which can enhance the sensitivity, selectivity, and conductivity of sensors. 2. They have good biocompatibility with biological tissues, which is conducive to the work of sensors in biological environments. 3. They can achieve the detection of trace substances.	1. The biocompatibility of some nanomaterials may have problems during long-term culture. 2. The preparation and modification processes of nanomaterials may be complex, increasing costs. 3. They may have stability issues in complex biological environments.

## References

[1] HogenEsch H., Nikitin A.Y. (2012). Challenges in Pre-Clinical Testing of Anti-Cancer Drugs in Cell Culture and in Animal Models. J. Control. Release.

[2] Paul S.M., Mytelka D.S., Dunwiddie C.T., Persinger C.C., Munos B.H., Lindborg S.R., Schacht A.L. (2010). How to improve R&D productivity: The pharmaceutical industry’s grand challenge. Nat. Rev. Drug Discov..

[3] Lancaster M.A., Knoblich J.A. (2014). Organogenesis in a dish: Modeling development and disease using organoid technologies. Science.

[4] Drost J., Clevers H. (2018). Organoids in cancer research. Nat. Rev. Cancer.

[5] Low L.A., Mummery C., Berridge B.R., Austin C.P., Tagle D.A. (2021). Organs-on-chips: Into the next decade. Nat. Rev. Drug Discov..

[6] Liang K.X. (2024). The application of brain organoid for drug discovery in mitochondrial diseases. Int. J. Biochem. Cell Biol..

[7] Singh D., Thakur A., Rakesh, Kumar A. (2025). Advancements in Organoid-Based Drug Discovery: Revolutionizing Precision Medicine and Pharmacology. Drug Dev. Res..

[8] Mujeeb U.R.M., Honarvar Nazari M., Sencan M. (2019). A novel semiconductor based wireless electrochemical sensing platform for chronic disease management. Biosens. Bioelectron..

[9] McDonald M., Sebinger D., Brauns L., Gonzalez-Cano L., Menuchin-Lasowski Y., Mierzejewski M., Psathaki O.E., Stumpf A., Wickham J., Rauen T. (2023). A mesh microelectrode array for non-invasive electrophysiology within neural organoids. Biosens. Bioelectron..

[10] Kim K., Lee Y., Jung K.B., Kim Y., Jang E., Lee M.O., Son M.Y., Lee H.J. (2025). Highly Stretchable 3D Microelectrode Array for Noninvasive Functional Evaluation of Cardiac Spheroids and Midbrain Organoids. Adv. Mater..

[11] Becker L., Janssen N., Layland S.L., Murdter T.E., Nies A.T., Schenke-Layland K., Marzi J. (2021). Raman Imaging and Fluorescence Lifetime Imaging Microscopy for Diagnosis of Cancer State and Metabolic Monitoring. Cancers.

[12] Dobric A., Tape C.J. (2025). High-dimensional signalling analysis of organoids. Curr. Opin. Cell Biol..

[13] Lin Z., Wang W., Liu R., Li Q., Lee J., Hirschler C., Liu J. (2025). Cyborg organoids integrated with stretchable nanoelectronics can be functionally mapped during development. Nat. Protoc..

[14] Zhou Y., Li C., Chen Y., Yu Y., Diao X., Chiu R., Fang J., Shen Y., Wang J., Zhu L. (2024). Human Airway Organoids and Multimodal Imaging-Based Toxicity Evaluation of 1-Nitropyrene. Environ. Sci. Technol..

[15] Hu X., Liu X., Xu Q., Ikkala O., Peng B. (2025). Mechanosensing of Stimuli Changes with Magnetically Gated Adaptive Sensitivity. ACS Mater. Lett..

[16] Kar E., Bose N., Dutta B., Mukherjee N., Mukherjee S. (2019). Ultraviolet- and Microwave-Protecting, Self-Cleaning e-Skin for Efficient Energy Harvesting and Tactile Mechanosensing. ACS Appl. Mater. Interfaces.

[17] Skardal A., Shupe T., Atala A. (2016). Organoid-on-a-chip and body-on-a-chip systems for drug screening and disease modeling. Drug Discov. Today.

[18] Rezaei Z., Wang N., Yang Y., Govindaraj K., Velasco J.J., Martinez Blanco A.D., Bae N.H., Lee H., Shin S.R. (2025). Enhancing organoid technology with carbon-based nanomaterial biosensors: Advancements, challenges, and future directions. Adv. Drug Deliv. Rev..

[19] Shin S.R., Kilic T., Zhang Y.S., Avci H., Hu N., Kim D., Branco C., Aleman J., Massa S., Silvestri A. (2017). Label-Free and Regenerative Electrochemical Microfluidic Biosensors for Continual Monitoring of Cell Secretomes. Adv. Sci..

[20] Cao U.M.N., Zhang Y., Chen J., Sayson D., Pillai S., Tran S.D. (2023). Microfluidic Organ-on-A-chip: A Guide to Biomaterial Choice and Fabrication. Int. J. Mol. Sci..

[21] Park S.E., Georgescu A., Huh D. (2019). Organoids-on-a-chip. Science.

[22] Sontheimer-Phelps A., Hassell B.A., Ingber D.E. (2019). Modelling cancer in microfluidic human organs-on-chips. Nat. Rev. Cancer.

[23] List M., Schmidt S., Christiansen H., Rehmsmeier M., Tan Q., Mollenhauer J., Baumbach J. (2016). Comprehensive analysis of high-throughput screens with HiTSeekR. Nucleic Acids Res..

[24] Cardoso B.D., Castanheira E.M.S., Lanceros-Mendez S., Cardoso V.F. (2023). Recent Advances on Cell Culture Platforms for In Vitro Drug Screening and Cell Therapies: From Conventional to Microfluidic Strategies. Adv. Healthc. Mater..

[25] Choi D., Gonzalez-Suarez A.M., Dumbrava M.G., Medlyn M., De Hoyos-Vega J.M., Cichocki F., Miller J.S., Ding L., Zhu M., Stybayeva G. (2024). Microfluidic Organoid Cultures Derived from Pancreatic Cancer Biopsies for Personalized Testing of Chemotherapy and Immunotherapy. Adv. Sci..

[26] Liu Z., Zhou Y., Lu J., Gong T., Ibanez E., Cifuentes A., Lu W. (2024). Microfluidic biosensors for biomarker detection in body fluids: A key approach for early cancer diagnosis. Biomark. Res..

[27] Homan K.A., Gupta N., Kroll K.T., Kolesky D.B., Skylar-Scott M., Miyoshi T., Mau D., Valerius M.T., Ferrante T., Bonventre J.V. (2019). Flow-Enhanced Vascularization and Maturation of Kidney Organoids in Vitro. Nat. Methods.

[28] Quintard C., Tubbs E., Jonsson G., Jiao J., Wang J., Werschler N., Laporte C., Pitaval A., Bah T.S., Pomeranz G. (2024). A Microfluidic Platform Integrating Functional Vascularized Organoids-on-Chip. Nat. Commun..

[29] Hu Y., Xing J., Zhang H., Pang X., Zhai Y., Cheng H., Xu D., Liao M., Qi Y., Wu D. (2024). Electroacoustic Responsive Cochlea-on-a-Chip. Adv. Mater..

[30] Tedjo W., Obeidat Y., Catandi G., Carnevale E., Chen T. (2021). Real-Time Analysis of Oxygen Gradient in Oocyte Respiration Using a High-Density Microelectrode Array. Biosensors.

[31] Gjorevski N., Sachs N., Manfrin A., Giger S., Bragina M.E., Ordonez-Moran P., Clevers H., Lutolf M.P. (2016). Designer matrices for intestinal stem cell and organoid culture. Nature.

[32] Debnath K., Heras K.L., Rivera A., Lenzini S., Shin J.W. (2023). Extracellular vesicle-matrix interactions. Nat. Rev. Mater..

[33] Liu Y., Sun L., Zhang H., Shang L., Zhao Y. (2021). Microfluidics for Drug Development: From Synthesis to Evaluation. Chem. Rev..

[34] Gough A., Soto-Gutierrez A., Vernetti L., Ebrahimkhani M.R., Stern A.M., Taylor D.L. (2021). Human biomimetic liver microphysiology systems in drug development and precision medicine. Nat. Rev. Gastroenterol. Hepatol..

[35] Elvira K.S. (2021). Microfluidic technologies for drug discovery and development: Friend or foe?. Trends Pharmacol. Sci..

[36] Wei F., Patel P., Liao W., Chaudhry K., Zhang L., Arellano-Garcia M., Hu S., Elashoff D., Zhou H., Shukla S. (2009). Electrochemical sensor for multiplex biomarkers detection. Clin. Cancer Res..

[37] Xiao F., Wang L., Duan H. (2016). Nanomaterial based electrochemical sensors for in vitro detection of small molecule metabolites. Biotechnol. Adv..

[38] Parihar A., Singhal A., Kumar N., Khan R., Khan M.A., Srivastava A.K. (2022). Next-Generation Intelligent MXene-Based Electrochemical Aptasensors for Point-of-Care Cancer Diagnostics. Nanomicro Lett..

[39] Kumar R., Singh R.K., Dubey P.K., Singh D.P., Yadav R.M. (2015). Self-Assembled Hierarchical Formation of Conjugated 3D Cobalt Oxide Nanobead-CNT-Graphene Nanostructure Using Microwaves for High-Performance Supercapacitor Electrode. ACS Appl. Mater. Interfaces.

[40] Maduraiveeran G., Sasidharan M., Ganesan V. (2018). Electrochemical sensor and biosensor platforms based on advanced nanomaterials for biological and biomedical applications. Biosens. Bioelectron..

[41] Brazys E., Ratautaite V., Mohsenzadeh E., Boguzaite R., Ramanaviciute A., Ramanavicius A. (2025). Formation of molecularly imprinted polymers: Strategies applied for the removal of protein template (review). Adv. Colloid Interface Sci..

[42] Sehit E., Drzazgowska J., Buchenau D., Yesildag C., Lensen M., Altintas Z. (2020). Ultrasensitive nonenzymatic electrochemical glucose sensor based on gold nanoparticles and molecularly imprinted polymers. Biosens. Bioelectron..

[43] Kukhta N.A., Marks A., Luscombe C.K. (2022). Molecular Design Strategies toward Improvement of Charge Injection and Ionic Conduction in Organic Mixed Ionic-Electronic Conductors for Organic Electrochemical Transistors. Chem. Rev..

[44] Bakhshandeh F., Zheng H., Barra N.G., Sadeghzadeh S., Ausri I., Sen P., Keyvani F., Rahman F., Quadrilatero J., Liu J. (2024). Wearable Aptalyzer Integrates Microneedle and Electrochemical Sensing for In Vivo Monitoring of Glucose and Lactate in Live Animals. Adv. Mater..

[45] Kim Y.J., Lee G.R., Cho E.N., Jung Y.S. (2020). Fabrication and Applications of 3D Nanoarchitectures for Advanced Electrocatalysts and Sensors. Adv. Mater..

[46] Aleman J., Kilic T., Mille L.S., Shin S.R., Zhang Y.S. (2021). Microfluidic integration of regeneratable electrochemical affinity-based biosensors for continual monitoring of organ-on-a-chip devices. Nat. Protoc..

[47] Hao R., Liu L., Yuan J., Wu L., Lei S. (2023). Recent Advances in Field Effect Transistor Biosensors: Designing Strategies and Applications for Sensitive Assay. Biosensors.

[48] Septiana W.L., Pawitan J.A. (2024). Potential Use of Organoids in Regenerative Medicine. Tissue Eng. Regen. Med..

[49] Bajgai J., Jun M., Oh J.H., Lee J.H. (2025). A perspective on the potential use of aptamer-based field-effect transistor sensors as biosensors for ovarian cancer biomarkers CA125 and HE4. Talanta.

[50] Yang S., Hu H., Kung H., Zou R., Dai Y., Hu Y., Wang T., Lv T., Yu J., Li F. (2023). Organoids: The current status and biomedical applications. MedComm.

[51] Wang H., Li X., Shi P., You X., Zhao G. (2024). Establishment and evaluation of on-chip intestinal barrier biosystems based on microfluidic techniques. Mater. Today Bio.

[52] Marcos L.F., Wilson S.L., Roach P. (2021). Tissue engineering of the retina: From organoids to microfluidic chips. J. Tissue Eng..

[53] Manimekala T., Sivasubramanian R., Dharmalingam G. (2022). Nanomaterial-Based Biosensors using Field-Effect Transistors: A Review. J. Electron. Mater..

[54] Hu W., Sheng Z., Hou X., Chen H., Zhang Z., Zhang D.W., Zhou P. (2021). Ambipolar 2D Semiconductors and Emerging Device Applications. Small Methods.

[55] Yang A.J., Wang S.-X., Xu J., Loh X.J., Zhu Q., Wang X.R. (2023). Two-Dimensional Layered Materials Meet Perovskite Oxides: A Combination for High-Performance Electronic Devices. ACS Nano.

[56] Tao J., Sun W., Lu L. (2022). Organic small molecule semiconductor materials for OFET-based biosensors. Biosens. Bioelectron..

[57] Chen S., Sun Y., Fan X., Xu Y., Chen S., Zhang X., Man B., Yang C., Du J. (2023). Review on two-dimensional material-based field-effect transistor biosensors: Accomplishments, mechanisms, and perspectives. J. Nanobiotechnol..

[58] Xiao W.-H., Hu Y., Yan K., Tang L.-M., Chen X., D’aGosta R., Yang K. (2025). Phonon-limited carrier mobility modeling of two-dimensional semiconductors based on first principles. J. Phys. Condens. Matter.

[59] Yu Z., Wang Q., Zeng T., Ye K., Zhou H., Han Z., Zeng Y., Fang B., Lv W., Geng L. (2025). Van der Waals Antiferroelectric CuCrP_2_S_6_-Based Artificial Synapse for High-Precision Neuromorphic Computation. Small.

[60] Huang J., Jiang Y., Ren Y., Liu Y., Wu X., Li Z., Ren J. (2020). Biomaterials and biosensors in intestinal organoid culture, a progress review. J. Biomed. Mater. Res. Part A.

[61] Otero J., Ulldemolins A., Farré R., Almendros I. (2021). Oxygen Biosensors and Control in 3D Physiomimetic Experimental Models. Antioxidants.

[62] Saorin G., Caligiuri I., Rizzolio F. (2022). Microfluidic organoids-on-a-chip: The future of human models. Semin. Cell Dev. Biol..

[63] Caipa Garcia A.L., Kucab J.E., Al-Serori H., Beck R.S.S., Fischer F., Hufnagel M., Hartwig A., Floeder A., Balbo S., Francies H. (2022). Metabolic Activation of Benzo (a) pyrene by Human Tissue Organoid Cultures. Int. J. Mol. Sci..

[64] Skottvoll F.S., Hansen F.A., Harrison S., Boger I.S., Mrsa A., Restan M.S., Stein M., Lundanes E., Pedersen-Bjergaard S., Aizenshtadt A. (2021). Electromembrane Extraction and Mass Spectrometry for Liver Organoid Drug Metabolism Studies. Anal. Chem..

[65] Dai C., Liu Y., Wei D. (2022). Two-Dimensional Field-Effect Transistor Sensors: The Road toward Commercialization. Chem. Rev..

[66] Nguyen T.T., Nguyen C.M., Huynh M.A., Vu H.H., Nguyen T.K., Nguyen N.T. (2023). Field effect transistor based wearable biosensors for healthcare monitoring. J. Nanobiotechnol..

[67] Li Y., Hu H., Shu J., Zhang G.J. (2025). Flexible Field-Effect Transistor Sensors for Next-Generation Health Monitoring: Materials to Advanced Applications. Small.

[68] Lv S., He E., Luo J., Liu Y., Liang W., Xu S., Zhang K., Yang Y., Wang M., Song Y. (2023). Using Human-Induced Pluripotent Stem Cell Derived Neurons on Microelectrode Arrays to Model Neurological Disease: A Review. Adv. Sci..

[69] Samarasinghe R.A., Miranda O.A., Buth J.E., Mitchell S., Ferando I., Watanabe M., Allison T.F., Kurdian A., Fotion N.N., Gandal M.J. (2021). Identification of neural oscillations and epileptiform changes in human brain organoids. Nat. Neurosci..

[70] Yang W., Ouyang Q., Zhu Z., Wu Y., Fan M., Liao Y., Guo X., Xu Z., Zhang X., Zhang Y. (2023). A biosensing system employing nonlinear dynamic analysis-assisted neural network for drug-induced cardiotoxicity assessment. Biosens. Bioelectron..

[71] Sharf T., van der Molen T., Glasauer S.M.K., Guzman E., Buccino A.P., Luna G., Cheng Z., Audouard M., Ranasinghe K.G., Kudo K. (2022). Functional neuronal circuitry and oscillatory dynamics in human brain organoids. Nat. Commun..

[72] Huang Z., Jia K., Tan Y., Yu Y., Xiao W., Zhou X., Yi J., Zhang C. (2025). Advances in cardiac organoid research: Implications for cardiovascular disease treatment. Cardiovasc. Diabetol..

[73] Luo Y., Song Y., Wang J., Xu T., Zhang X. (2024). Integrated Mini-Pillar Platform for Wireless Real-Time Cell Monitoring. Research.

[74] Volmert B., Kiselev A., Juhong A., Wang F., Riggs A., Kostina A., O’Hern C., Muniyandi P., Wasserman A., Huang A. (2023). A patterned human primitive heart organoid model generated by pluripotent stem cell self-organization. Nat. Commun..

[75] Martinelli E., Akouissi O., Liebi L., Furfaro I., Maulà D., Savoia N., Remy A., Nikles L., Roux A., Stoppini L. (2024). The E-Flower: A Hydrogel-Actuated 3D MEA for Brain Spheroid Electrophysiology. Sci. Adv..

[76] Yang X., Forró C., Li T.L., Miura Y., Zaluska T.J., Tsai C.-T., Kanton S., McQueen J.P., Chen X., Mollo V. (2024). Kirigami Electronics for Long-Term Electrophysiological Recording of Human Neural Organoids and Assembloids. Nat. Biotechnol..

[77] Didier C.M., Fox D., Pollard K.J., Baksh A., Iyer N.R., Bosak A., Li Sip Y.Y., Orrico J.F., Kundu A., Ashton R.S. (2023). Fully Integrated 3D Microelectrode Arrays with Polydopamine-Mediated Silicon Dioxide Insulation for Electrophysiological Interrogation of a Novel 3D Human, Neural Microphysiological Construct. ACS Appl. Mater. Interfaces.

[78] Xu Q., Wang L., Xi Y., Ruan T., Cao J., Xu M., Zheng K., Du Z., Wei N., Wang X. (2025). An Efficient MEMS Microelectrode Array with Reliable Interelectrode Insulation Processes for In Vivo Neural Recording. Small.

[79] Liu Y., Hu Q., Yang X., Kang J. (2024). Unveiling the potential of amorphous nanocatalysts in membrane-based hydrogen production. Mater. Horiz..

[80] Xia Q., Liu R., Chen X., Chen Z., Zhu J.J. (2023). In Vivo Voltammetric Imaging of Metal Nanoparticle-Catalyzed Single-Cell Electron Transfer by Fermi Level-Responsive Graphene. Research.

[81] Hang X., He S., Dong Z., Minnick G., Rosenbohm J., Chen Z., Yang R., Chang L. (2021). Nanosensors for single cell mechanical interrogation. Biosens. Bioelectron..

[82] Beech D.J., Kalli A.C. (2019). Force Sensing by Piezo Channels in Cardiovascular Health and Disease. Arter. Thromb. Vasc. Biol..

[83] Wang J., Chen X., Li R., Wang S., Geng Z., Shi Z., Jing Y., Xu K., Wei Y., Wang G. (2025). Standardization and consensus in the development and application of bone organoids. Theranostics.

[84] Sato T. (2025). Role of Patient-Derived Tumor Organoids in Advanced Cancer Research. J. Nippon. Med. Sch..

[85] Abdel Fattah A.R., Daza B., Rustandi G., Berrocal-Rubio M.A., Gorissen B., Poovathingal S., Davie K., Barrasa-Fano J., Condor M., Cao X. (2021). Actuation enhances patterning in human neural tube organoids. Nat. Commun..

[86] Tian Y., Hou L.X., Zhang X.N., Du M., Zheng Q., Wu Z.L. (2024). Engineering Tough Supramolecular Hydrogels with Structured Micropillars for Tunable Wetting and Adhesion Properties. Small.

[87] Cortelli G., Grob L., Patruno L., Cramer T., Mayer D., Fraboni B., Wolfrum B., de Miranda S. (2023). Determination of Stiffness and the Elastic Modulus of 3D-Printed Micropillars with Atomic Force Microscopy-Force Spectroscopy. ACS Appl. Mater. Interfaces.

[88] Fajrial A.K., Liu K., Gao Y., Gu J., Lakerveld R., Ding X. (2021). Characterization of Single-Cell Osmotic Swelling Dynamics for New Physical Biomarkers. Anal. Chem..

[89] Tang Z.X., Wang B., Li Z.R., Huang Z., Zhao H.X., Long L.S., Zheng L.S. (2024). Enhancing the performance of molecule-based piezoelectric sensors by optimizing their microstructures. Chem. Sci..

[90] Lyu Q., Gong S., Lees J.G., Yin J., Yap L.W., Kong A.M., Shi Q., Fu R., Zhu Q., Dyer A. (2022). A soft and ultrasensitive force sensing diaphragm for probing cardiac organoids instantaneously and wirelessly. Nat. Commun..

[91] Shi Q., Sun Z., Le X., Xie J., Lee C. (2023). Soft Robotic Perception System with Ultrasonic Auto-Positioning and Multimodal Sensory Intelligence. ACS Nano.

[92] Xiao Z., Ren Z., Zhuge Y., Zhang Z., Zhou J., Xu S., Xu C., Dong B., Lee C. (2024). Multimodal In-Sensor Computing System Using Integrated Silicon Photonic Convolutional Processor. Adv. Sci..

[93] Guo F., Li Y., Ma G., Zhang M., Fu J., Luo C., Yuan L., Long Y. (2024). Overview of 3D Printing Multimodal Flexible Sensors. ACS Appl. Mater. Interfaces.

[94] Maly P., Brixner T. (2021). Fluorescence-Detected Pump-Probe Spectroscopy. Angew. Chem. Int. Ed. Engl..

[95] Yu Q., Yao Z., Zhang H., Li Z., Chen Z., Xiong H. (2023). Transient Stimulated Raman Excited Fluorescence Spectroscopy. J. Am. Chem. Soc..

[96] Qian N., Xiong H., Wei L., Shi L., Min W. (2025). Merging Vibrational Spectroscopy with Fluorescence Microscopy: Combining the Best of Two Worlds. Annu. Rev. Phys. Chem..

[97] Ansaryan S., Chiang Y.C., Liu Y.C., Tan J., Lorenzo-Martin L.F., Lutolf M.P., Tolstonog G., Altug H. (2025). Spatiotemporal Interrogation of Single Spheroids Using Multiplexed Nanoplasmonic-Fluorescence Imaging. Small Methods.

[98] Huang K., Li M., Li Q., Chen Z., Zhang Y., Gu Z. (2024). Image-based profiling and deep learning reveal morphological heterogeneity of colorectal cancer organoids. Comput. Biol. Med..

[99] Luo X., Chen M., Shan H., Yu X., Lin Q., Tao Q., Wei X., Lv C., Chen Z., Zhuo F. (2025). Label-Free 3D Photoacoustic Imaging of Tumor Organoids for Volumetric Drug Screening. Adv. Sci..

[100] Fatima M., Abbas N. (2025). Expanding Horizons in Advancements of FRET Biosensing Technologies. Biosensors.

[101] Tirgar P., Vikstrom A., Sepulveda J.M.R., Srivastava L.K., Amini A., Tabata T., Higo S., Bub G., Ehrlicher A. (2025). Heart-on-a-Miniscope: A Miniaturized Solution for Electrophysiological Drug Screening in Cardiac Organoids. Small.

[102] Barth B.M., Sharma R., Altinoglu E.I., Morgan T.T., Shanmugavelandy S.S., Kaiser J.M., McGovern C., Matters G.L., Smith J.P., Kester M. (2010). Bioconjugation of calcium phosphosilicate composite nanoparticles for selective targeting of human breast and pancreatic cancers in vivo. ACS Nano.

[103] Chandra A., Kumar V., Garnaik U.C., Dada R., Qamar I., Goel V.K., Agarwal S. (2024). Unveiling the Molecular Secrets: A Comprehensive Review of Raman Spectroscopy in Biological Research. ACS Omega.

[104] Wang J., Chen M., Zhao X., Wang Y., Li D. (2024). Fourier Raman light field microscopy based on surface-enhanced Raman scattering. Opt. Lett..

[105] Kralova K., Kral M., Vrtelka O., Setnicka V. (2024). Comparative study of Raman spectroscopy techniques in blood plasma-based clinical diagnostics: A demonstration on Alzheimer’s disease. Spectrochim. Acta Part A Mol. Biomol. Spectrosc..

[106] Bell S.E.J., Charron G., Cortes E., Kneipp J., de la Chapelle M.L., Langer J., Prochazka M., Tran V., Schlucker S. (2020). Towards Reliable and Quantitative Surface-Enhanced Raman Scattering (SERS): From Key Parameters to Good Analytical Practice. Angew. Chem. Int. Ed. Engl..

[107] Lian S., Li X., Lv X. (2025). Recent Developments in SERS Microfluidic Chips: From Fundamentals to Biosensing Applications. ACS Appl. Mater. Interfaces.

[108] Skinner W.H., Robinson N., Hardisty G.R., Fleming H., Geddis A., Bradley M., Gray R.D., Campbell C.J. (2023). SERS microsensors for pH measurements in the lumen and ECM of stem cell derived human airway organoids. Chem. Commun..

[109] Mou L., Mandal K., Mecwan M.M., Hernandez A.L., Maity S., Sharma S., Herculano R.D., Kawakita S., Jucaud V., Dokmeci M.R. (2022). Integrated biosensors for monitoring microphysiological systems. Lab Chip.

[110] Abdelhamid H.N., Wu H.F. (2013). Multifunctional graphene magnetic nanosheet decorated with chitosan for highly sensitive detection of pathogenic bacteria. J. Mater. Chem. B.

[111] Ma L., Yang X., Xue S., Zhou R., Wang C., Guo Z., Wang Y., Cai J. (2025). “Raman plus X” dual-modal spectroscopy technology for food analysis: A review. Compr. Rev. Food Sci. Food Saf..

[112] Lauwerends L.J., Abbasi H., Bakker Schut T.C., Van Driel P., Hardillo J.A.U., Santos I.P., Barroso E.M., Koljenovic S., Vahrmeijer A.L., Baatenburg de Jong R.J. (2022). The complementary value of intraoperative fluorescence imaging and Raman spectroscopy for cancer surgery: Combining the incompatibles. Eur. J. Nucl. Med. Mol. Imaging.

[113] Reuter C., Hauswald W., Burgold-Voigt S., Hubner U., Ehricht R., Weber K., Popp J. (2024). Imaging Diffractometric Biosensors for Label-Free, Multi-Molecular Interaction Analysis. Biosensors.

[114] Hanifa Lestari T.F., Irkham I., Pratomo U., Gaffar S., Zakiyyah S.N., Rahmawati I., Topkaya S.N., Hartati Y.W. (2024). Label-free and label-based electrochemical detection of disease biomarker proteins. ADMET DMPK.

[115] Zhou C., Wu Y., Wang Z., Liu Y., Yu J., Wang W., Chen S., Wu W., Wang J., Qian G. (2023). Standardization of organoid culture in cancer research. Cancer Med..

[116] Liu X., Zhou Z., Zhang Y., Zhong H., Cai X., Guan R. (2025). Recent progress on the organoids: Techniques, advantages and applications. Biomed. Pharmacother..

[117] Hu C., He S., Lee Y.J., He Y., Kong E.M., Li H., Anastasio M.A., Popescu G. (2022). Live-dead assay on unlabeled cells using phase imaging with computational specificity. Nat. Commun..

[118] Zhang Y., Li T., Tao H., Liu F., Hu B., Wu M., Yu H. (2023). Research on adaptive impedance control technology of upper limb rehabilitation robot based on impedance parameter prediction. Front. Bioeng. Biotechnol..

[119] Duan Q., Liu Y., Chang S., Chen H., Chen J.H. (2021). Surface Plasmonic Sensors: Sensing Mechanism and Recent Applications. Sensors.

[120] He Z., Li F., Zuo P., Tian H. (2023). Principles and Applications of Resonance Energy Transfer Involving Noble Metallic Nanoparticles. Materials.

[121] Bankoglu Yola B., Ozdemir N., Yola M.L. (2024). A Review Study on Molecularly Imprinting Surface Plasmon Resonance Sensors for Food Analysis. Biosensors.

[122] Springer T., Bockova M., Slaby J., Sohrabi F., Capkova M., Homola J. (2025). Surface plasmon resonance biosensors and their medical applications. Biosens. Bioelectron..

[123] Zemaitis K.J., Lin V.S., Ahkami A.H., Winkler T.E., Anderton C.R., Velickovic D. (2023). Expanded Coverage of Phytocompounds by Mass Spectrometry Imaging Using On-Tissue Chemical Derivatization by 4-APEBA. Anal. Chem..

[124] Guan X., Lu Q., Liu S., Yan X. (2024). Postionization Mass Spectrometry Imaging: Past, Present, and Future. Mass Spectrom. Rev..

[125] Dayanidhi D.L., Watlington W.K., Mantyh J.B., Rupprecht G., Hsu D.S. (2023). Effects and Eradication of Mycoplasma Contamination on Patient-derived Colorectal Cancer Organoid Cultures. Cancer Res. Commun..

[126] Zhao H., Cheng Y., Li J., Zhou J., Yang H., Yu F., Yu F., Khutsishvili D., Wang Z., Jiang S. (2024). Droplet-engineered organoids recapitulate parental tissue transcriptome with inter-organoid homogeneity and inter-tumor cell heterogeneity. Fundam. Res..

[127] Matsumoto M., Morimoto Y., Sato T., Takeuchi S. (2022). Microfluidic Device to Manipulate 3D Human Epithelial Cell-Derived Intestinal Organoids. Micromachines.

[128] Wang M., Zhou X., Zhou S., Wang M., Jiang J., Wu W., Liu T., Xu W., Zhang J., Liu D. (2023). Mechanical force drives the initial mesenchymal-epithelial interaction during skin organoid development. Theranostics.

[129] Ronzetti M., Simeonov A. (2025). A comprehensive update on the application of high-throughput fluorescence imaging for novel drug discovery. Expert Opin. Drug Discov..

[130] Ma Z., Liu Y., Chen R., Fan H., Kong L., Cao X. (2025). A novel perspective on bone tumors: Advances in organoid research. Front. Pharmacol..

[131] El-Sewify I.M., Shenashen M.A., El-Agamy R.F., Emran M.Y., Selim M.S., Khairy M., Shahat A., Selim M.M., Elmarakbi A., Ebara M. (2024). Fluorescent sensor/tracker for biocompatible and real-time monitoring of ultra-trace arsenic toxicants in living cells. J. Hazard. Mater..

[132] Abugomaa A., Elbadawy M., Yamanaka M., Goto Y., Hayashi K., Mori T., Uchide T., Azakami D., Fukushima R., Yoshida T. (2020). Establishment of 2.5D organoid culture model using 3D bladder cancer organoid culture. Sci. Rep..

[133] Jiang X., Oyang L., Peng Q., Liu Q., Xu X., Wu N., Tan S., Yang W., Han Y., Lin J. (2023). Organoids: Opportunities and challenges of cancer therapy. Front. Cell Dev. Biol..

[134] Song J., Luo Y., Hao Z., Qu M., Huang C., Wang Z., Yang J., Liang Q., Jia Y., Song Q. (2025). Graphene-based wearable biosensors for point-of-care diagnostics: From surface functionalization to biomarker detection. Mater. Today Bio.

[135] Huang Y., Zhang X., Zhang W., Tang J., Liu J. (2025). Rational design matrix materials for organoid development and application in biomedicine. Regen. Biomater..

[136] Narazaki G., Miura Y., Pavlov S.D., Thete M.V., Roth J.G., Avar M., Shin S., Kim J.I., Hudacova Z., Heilshorn S.C. (2025). Scalable production of human cortical organoids using a biocompatible polymer. Nat. Biomed. Eng..

[137] Zhong G., Liu Q., Wang Q., Qiu H., Li H., Xu T. (2024). Fully integrated microneedle biosensor array for wearable multiplexed fitness biomarkers monitoring. Biosens. Bioelectron..

[138] Chimene D., Saleem W., Longbottom N., Ko B., Jeevarathinam A.S., Horn S., McShane M.J. (2024). Long-Term Evaluation of Inserted Nanocomposite Hydrogel-Based Phosphorescent Oxygen Biosensors: Evolution of Local Tissue Oxygen Levels and Foreign Body Response. ACS Appl. Bio Mater..

[139] Xue J., Chu Y., Huang Y., Chen M., Sun M., Fan Z., Wu Y., Chen L. (2024). A tumorigenicity evaluation platform for cell therapies based on brain organoids. Transl. Neurodegener..

[140] Cooper R.M., Wright J.A., Ng J.Q., Goyne J.M., Suzuki N., Lee Y.K., Ichinose M., Radford G., Ryan F.J., Kumar S. (2023). Engineered bacteria detect tumor DNA. Science.

[141] Pang X., Hu Y., Dai Z., Lou Q., Xu W., Chen L. (2025). Precision medicine research progress based on colorectal cancer organoids. Discov. Oncol..

[142] Motoike S., Inada Y., Toguchida J., Kajiya M., Ikeya M. (2025). Jawbone-like organoids generated from human pluripotent stem cells. Nat. Biomed. Eng..

[143] Li Y.X., Zhang S., Huang Y., Li J., Chen Y., Gao L., Dai H. (2025). Portable multimodal platform with carbon nano-onions as colorimetric and fluorescent signal output for trypsin detection. Talanta.

